# Structure-function analyses of candidate small molecule RPN13 inhibitors with antitumor properties

**DOI:** 10.1371/journal.pone.0227727

**Published:** 2020-01-15

**Authors:** Ravi K. Anchoori, Marietta Tan, Ssu-Hsueh Tseng, Shiwen Peng, Ruey-Shyang Soong, Aliyah Algethami, Palmer Foran, Samarjit Das, Chenguang Wang, Tian-Li Wang, Hong Liang, Chien-Fu Hung, Richard B. S. Roden

**Affiliations:** 1 Department of Pathology, The Johns Hopkins University, Baltimore, Maryland, United States of America; 2 Department of Oncology, The Johns Hopkins University, Baltimore, Maryland, United States of America; 3 Department of Otolaryngology, The Johns Hopkins University, Baltimore, Maryland, United States of America; 4 Department of Anesthesiology and Critical Care Medicine, The Johns Hopkins University, Baltimore, Maryland, United States of America; 5 Department of Biostatistics, The Johns Hopkins University, Baltimore, Maryland, United States of America; 6 Department of Gynecology and Obstetrics, The Johns Hopkins University, Baltimore, Maryland, United States of America; University of Florida, UNITED STATES

## Abstract

We sought to design ubiquitin-proteasome system inhibitors active against solid cancers by targeting ubiquitin receptor RPN13 within the proteasome’s 19S regulatory particle. The prototypic bis-benzylidine piperidone-based inhibitor RA190 is a michael acceptor that adducts Cysteine 88 of RPN13. In probing the pharmacophore, we showed the benefit of the central nitrogen-bearing piperidone ring moiety compared to a cyclohexanone, the importance of the span of the aromatic wings from the central enone-piperidone ring, the contribution of both wings, and that substituents with stronger electron withdrawing groups were more cytotoxic. Potency was further enhanced by coupling of a second warhead to the central nitrogen-bearing piperidone as RA375 exhibited ten-fold greater activity against cancer lines than RA190, reflecting its nitro ring substituents and the addition of a chloroacetamide warhead. Treatment with RA375 caused a rapid and profound accumulation of high molecular weight polyubiquitinated proteins and reduced intracellular glutathione levels, which produce endoplasmic reticulum and oxidative stress, and trigger apoptosis. RA375 was highly active against cell lines of multiple myeloma and diverse solid cancers, and demonstrated a wide therapeutic window against normal cells. For cervical and head and neck cancer cell lines, those associated with human papillomavirus were significantly more sensitive to RA375. While *ARID1A*-deficiency also enhanced sensitivity 4-fold, RA375 was active against all ovarian cancer cell lines tested. RA375 inhibited proteasome function in muscle for >72h after single *i*.*p*. administration to mice, and treatment reduced tumor burden and extended survival in mice carrying an orthotopic human xenograft derived from a clear cell ovarian carcinoma.

## Introduction

The eukaryotic 26S proteasome is a 2.5MDa complex of 47 proteins arranged in a symmetrical barrel of two central 20S catalytic subunits (CS) capped at either end by 19S regulatory particles (RP) that act together to mediate targeted degradation of proteins via a coordinated multi-step process. Proteins are targeted for proteasomal degradation by coupling of ubiquitin in extended K48-linked chains via a ubiquitin ligase. Proteins displaying K48-linked ubiquitin chains are first recognized by 19S RP, unfolded and deubiquitinated therein, and subsequently passed on to the catalytic 20S core particle for degradation into small peptides by its three distinct proteolytic active sites [1]. Although the proteasome performs key homeostatic cellular functions, targeted inhibition of the 20S proteasome chymotryptic catalytic subunit PSMB5 is used for the treatment of multiple myeloma (MM) and mantle cell lymphoma (MCL), and three such inhibitors have been licensed (bortezomib, ixazomib and carfilzomib). However, to date, they have failed to demonstrate efficacy against solid tumors, possibly reflecting limited drug assess, and MM and MCL patients frequently develop resistant disease and treatment-related peripheral neuropathy [2, 3]. Thus, there is considerable interest in addressing the limitations associated with 20S proteasome inhibitors by targeting upstream proteasome activities in the 19S RP including substrate recognition, deubiquitination enzymes (DUBs) and/or ATP-dependent unfolding [4].

Two ubiquitin receptors, RPN13 and RPN10 in the 19S RP act in concert to recognize protein substrates tagged with K48-linked chains of ≥4 ubiquitin subunits. In addition to its ubiquitin binding Pru N-terminal domain [5], RPN13 also binds the deubiquitinase (DUB) UCH37/UCHL5, and RPN2 recruiting it to the 19S RP [6, 7]. RPN13 interaction also enhances the DUB activity of UCH37, likely by opening up its active site [8, 9]. This DUB activity allows recycling of the ubiquitin subunits and, after ATP-dependent unfolding, progression of the substrate to the 20S catalytic subunit for degradation. RA190 was identified as an RPN13 inhibitor that blocks proteasome-mediated deubiquitination and proteolysis [6]. Although binding to UCH37 can also occur *in vitro* [6], several lines of evidence including cell labeling [10], degrader [11] and knockout studies [12] suggest that RPN13 is RA190’s principle cellular target. RA190 and the related RA183 [13] exhibit antineoplastic activity, including against bortezomib-resistant MM, and against solid tumors in pre-clinical models of ovarian cancer [10, 13, 14], human papillomavirus (HPV)-associated and several other solid cancers [6, 12, 15–18]. Like RA190 and RA183 [13], the diphenyldihaloketones CLEFMA and EF-24 also adduct to RPN13 and inhibit proteasome function [19], and herein we seek to further understand the pharmacophore. Ovarian cancer is a promising target because RPN13, which is encoded by *ADRM1*, is consistently over-expressed in high grade serous carcinoma, and this occurs in the precursor lesion [17]. Amplification of *ADRM1* is most common in ovarian cancer and is associated with shorter time to recurrence and overall survival [20, 21], although it does not predict sensitivity to RA190 in cell lines [17]. Ectopic *ADRM1* overexpression in cell lines increases proliferation, migration, and growth in soft agar, and knock-down of *ADRM1* expression results in apoptosis, and it has been suggested as an oncogene and novel therapeutic target for ovarian cancer [21].

Chronic accumulation of misfolded polyubiquitinated proteins is associated with ovarian cancer, and it is exacerbated by proteasome inhibition [22, 23]. The Unfolded Protein Response (UPR) is triggered in an effort to restore proteostasis and relieve endoplasmic reticulum stress, and if unresolved, leads to p53-independent apoptosis [24, 25]. Elevated generation of intracellular reactive oxygen species (ROS) is frequently associated with malignant transformation due to oncogene activation and/or enhanced metabolism in tumor cells and confers oxidative injury. This potentially provides a therapeutic window and treatment with proteasome inhibitor bortezomib rapidly induces oxidative stress that is considered a major contributor to its anticancer properties [26, 27].

Because of the potential for competition with, or inactivation by, cellular glutathione, we also tested incorporation of an additional warhead, including chloroacetamide, intended to lower cellular glutathione levels [28, 29], thereby limiting inactivation of our chalcone-based RPN13 inhibitors and increasing oxidative stress and their antitumor potency.

## Materials and methods

### Cell lines and cytotoxicity assays

All cell lines were obtained from the American Type Culture Collection (ATCC) and cultured in the specified medium supplemented with 10% fetal bovine serum, 100 IU/mL penicillin, and 100 μg/mL streptomycin at 37°C in a humidified 5% CO_2_/95% air incubator. Synthesis of key compounds is described in **S1 Methods**, and >97% purity of RA375 was confirmed by NMR and MS. To assess drug cytotoxicity cells were seeded at 2,500 cells/well (10,000 cells/well for MM lines) in 100 μL medium in 96-well plate. The cells were treated with compounds for 48h, cells were incubated according to the manufacturer’s protocol with the Thiazolyl Blue Tetrazolium Bromide (Sigma, M2128) and A_570_ measured using a Benchmark Plus microplate spectrophotometer (BIO-RAD).

### Antibodies and Western Blot Analyses

Cell lysate (10–20 μg total protein) prepared in MPER (Pierce) from each sample was subjected to SDS-PAGE, transferred to PVDF membranes and analyzed by Western blot using antibodies specific for ubiquitin (P4D1, sc-8017, Santa Cruz), actin (#66009, Protein Tech Group), Lys48-linkled ubiquitin (Apu 2, 05–1307, (Millipore), caspase-3 (51-68655X, BD Pharmingen) at the dilutions recommended by the manufacturers, and for secondary antibodies we utilized either peroxidase-linked anti-mouse IgG or anti-rabbit IgG (GE Healthcare UK Ltd), HRP conjugated streptavidin (N100, Thermo Fisher) at the recommended dilution. The blots were developed using HyGLO chemiluminescent detection reagent (Denville).

### Flow cytometry analysis of cell death and ROS

10^5^ cells were re-suspended in binding buffer, 5 μL of Annexin V-PE (Apoptosis Detection Kit I (BD Pharmingen) and 5 μL of 7-AAD were then added into the cells, which were then incubated at room temperature for 15 minutes and analyzed by flow cytometry on a FACSCalibur using CellQuest software (Becton Dickinson). Active caspase-3 was measured by flow cytometry using PE-Active Caspase-3 antibody (550821) and 1X BD cytofix/cytoperm fixation buffer (51-6896kc) (BD Pharmingen) according to the manufacturer’s protocol. For assay of ROS, 2 x 10^5^ cells were plated in a 6-well plate the day before treatment with compounds or vehicle. After treatment, plates were washed once with PBS, and the cells were harvested using trypsin-EDTA. Cells were washed again with PBS and then suspended in 1 mL of PBS and incubated with 1 μM 2,7-dichlorofluorescein diacetate (H_2_DCFDA) at 37°C for 60 min. Cells were then washed twice with PBS and analyzed by flow cytometry.

### RA190B labeling assay

Clarified cell lysate in MPER buffer was pretreated with streptavidin magna beads for 45 min at 4°C to deplete non-specific biotinylated proteins in the cell lysate. The beads were separated and 40 μL of the pre-cleared cell lysate was incubated with compounds (20 μM) for 45 min at 4°C, and then treated with RA190B (10 μM) for 45 min at 4°C. Next, the samples were mixed with Laemmli sample buffer (BioRad) and boiled for 5 min. The proteins were separated using a 4–15% Bio-Rad Mini-PROTEAN SDS-PAGE gel (1 hr at 100 V), and transferred to PVDF membrane overnight at 4°C (24 V). The membrane was blocked with 5% BSA in PBST for 1 hr at RT and washed for 20 minutes (3X with PBST). Then the membrane was probed with HRP-streptavidin (1:10,000 in PBST) for 1 hr at RT, washed for 30 min (3X with PBST), and developed using HyGLO chemiluminescent detection reagent (Denville) for biotin detection.

### Luciferase and GSH assays

Sub-confluent cultures of cells were transfected with 4Ub-FL or FL plasmid using Lipofectamine 2000 reagent (Life Technologies). Cells were seeded at 10,000 cells/well in 96-wells plate 48 hr post transfection and incubated with compounds or vehicle (DMSO) at the doses and times indicated. Luciferase activity in cell lysate was determined with a luciferase assay kit (Promega) according to the manufacturer’s instructions. Bioluminescence was measured by using a Glomax Multidetection system (Promega). Glutathione was assayed using the Promega GSH assay kit (V6611).

### Q-PCR to measure mRNA levels

Total RNA was isolated from cells using the RNeasy mini kit (Qiagen) according to the manufacturer’s instructions. Extracted RNA was normalized for concentration and reverse transcribed using an iScript cDNA synthesis kit (Bio-Rad) according to the manufacturer’s instructions. *CHOP10* expression levels were measured by Taqman gene expression assays with Taqman gene expression master mix (Applied Biosystems) and run with a standard thermal cycling protocol. Spliced *XBP1* mRNA was assayed with SsoFast EvaGreen Supermix (Bio-rad) following the protocol for the iCycler System using as primers: F: 5’-TGCTGAGTCCGCAGCAGGTG-3’ and R: 5’-TGGGTCCAAGTTGTCCAGAATGCC-3’. Calculations were done according to the Livak method and normalized to the reference gene GAPDH. Each condition was replicated three times; each sample was run in triplicate.

### Animal studies

All animal procedures were performed according to protocols approved by the Johns Hopkins University Animal Care and Use Committee, and in accordance with the AAALAC recommendations for the proper use and care of laboratory animals (protocol MO15M375, renewed as MO18M129). Four to six week old female Nude (002019), or Balb/c (000651) mice were purchased from Jackson Laboratories (ME, USA). Isoflurane anesthesia was used during imaging. The health conditions and/or criteria under which early euthanasia or withdrawal of an animal from the study was implemented included, but are not limited to, general signs of distress such as hunched posture, lethargy, anorexia, dehydration, rough hair coat and/or those that are directly related to the experimental procedures e.g. loss of weight >10%, lethargy, restricted movement of limbs, distended abdomen. Animals in distress were euthanized by carbon dioxide asphyxiation, and cervical dislocation was used to ensure death. This is an acceptable form of euthanasia for mice and in compliance with the recommendations of the Panel on Euthanasia of the American Veterinary Medical Association.

### Electroporation method

A patch of Balb/c mouse leg was shaved of hair and 10 μg 4Ub-FL plasmid in 20 μL of PBS was injected into the *quadriceps femoralis* muscle followed immediately by injection of the 2 Needle Array to 5 mm depth encompassing the injection site and square wave electroporation (ElectroSquarePorator 833, BTX-2 Needle array 5mm gap, Harvard apparatus) delivered as eight pulses at 106V for 20 ms with 200 ms intervals. One day post electroporation, mice were anesthetized with isoflurane, injected i.p. with luciferin (0.3 mg in 100 μL water) and optical imaging was performed to determine basal level luciferase expression. Images were acquired for 10 min with a Xenogen IVIS 200 (Caliper, Hopkinton, MA). Equally sized areas were analyzed using Living Image 2.20 software. Mice were imaged weekly during treatment.

### Tumor studies

Nude mice were inoculated with 10^6^ ES2-Luciferase cells *i*.*p*. in 100 μL PBS. At day 3, mice were imaged for basal level luminescence activity with a Xenogen IVIS 200 after injection *i*.*p*. with luciferin (0.3 mg in 100 μL water). Mice were randomized into two groups (n = 8) and treated *i*.*p*. with 10mg/Kg RA375 or vehicle (25% (*w*/*v*) β-Hydroxypropylcyclodextrin in water), and imaged again on days 7 and 14.

### Statistical analyses

Results are reported as mean ± standard deviation (s.d.). Statistical significance of differences was assessed by ordinary 1-way ANOVA using Tukey’s multiple comparison test in Prism (V.8.2.0 Graphpad, San Diego, CA) to correct for the false discovery rate with the level of significance set at p≤0.05. Survival was summarized using Kaplan–Meier methods and compared using log-rank tests. Combination indices (CI) were calculated using Synergy finder [30].

## Results

**Fig 1:**
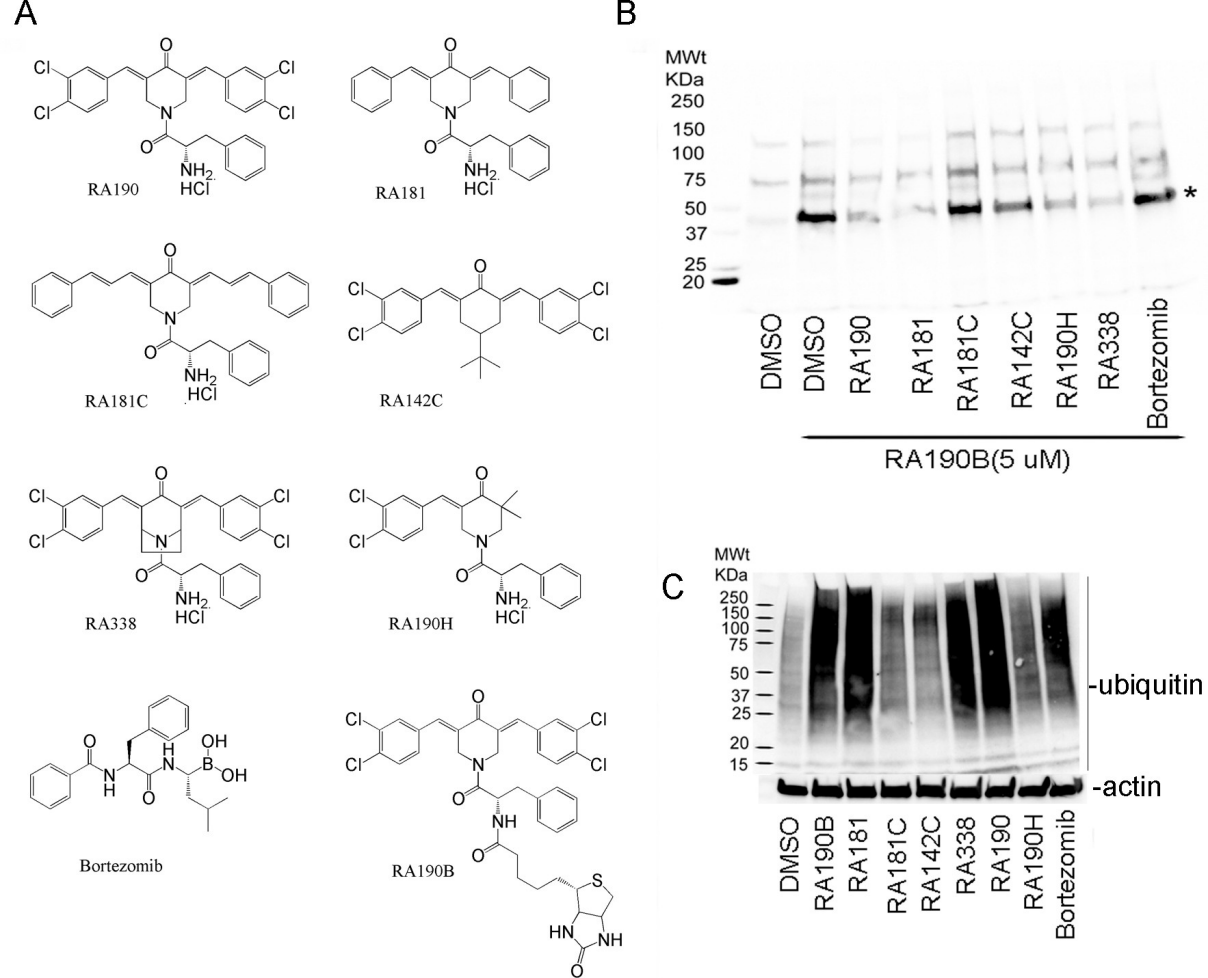
Probing requirements to bind a 42kDa cellular target and accumulate high molecular weight polyubiquitinated proteins. **(A)** Chemical structures of candidate inhibitors. **(B)** Precleared ES2 cell lysate was incubated with compounds (20 μM) or vehicle (DMSO, 1:100) for 45 min and then with RA190B (5 μM) for 45 min at 4°C. Samples were subjected to SDS-PAGE, transfer to PVDF membrane, and after probing with HRP-streptavidin, binding was detected using chemiluminescence. **(C)** ES2 cells were treated with compounds (1 μM, 4 hr), lysed and the samples probed with ubiquitin or actin-specific antibody by Western blot.

To further elucidate the pharmacophore and minimal structural features necessary for RPN13 binding and antineoplastic activity, we synthesized a new series of compounds based upon bis-benzylidine ring moieties with various modifications in and around the ring (**S1 Table**). Biotinylated RA190 (RA190B, **Fig 1A**) is utilized as a probe to detect covalent binding to its cellular target(s) [10] in detergent lysates of cancer cell lines. Using this approach a predominant band of 42KDa was labeled by RA190B and subsequently identified as RPN13 [10]. Here, we examined the ability of compounds (**Fig 1A**) to compete RA190B binding to its 42kDa cellular target (**Fig 1B**). Compounds, including bortezomib as a negative control, were incubated with HeLa cell lysate and then RA190B added. Upon SDS-PAGE, transfer to PVDF membrane and probing these lysates with HRP-streptavidin it was evident that nitrogen-bearing piperidone ring moieties (RA190, RA181, RA190H, and RA338) competed labeling by RA190B to a greater extent compared to a cyclohexanone bis-benzylidine moiety, RA142C. The ability of RA181, but not RA181C, to compete RA190B binding to the 42kDa cellular target suggests that the span of the aromatic wings is an important factor for RPN13 binding (**Fig 1B**). The half molecule RA190H did compete RA190B labeling, suggesting that a single aromatic wing and enone suffice. However, the IC_50_ for killing HeLa cells by RA190H is 6-fold higher than for RA190, 562 nM versus 85 nM respectively (**S1 Table**), and it only weakly caused an accumulation of high molecular weight polyubiquitinated proteins (**Fig 1C**), suggesting both are optimal. The compounds which competed binding of RA190B to its 42kDa cellular target, RA181 and RA338, also produced a rapid accumulation of high molecular weight polyubiquitinated proteins when added to cells. Conversely, RA181C, RA142C, and RA181C, which failed to compete binding by RA190B, produced only minimal accumulation of polyubiquitinated proteins (**Fig 1C**) and exhibited >2500 nM IC_50_ in cell killing assays (**S1 Table**).

**Fig 2:**
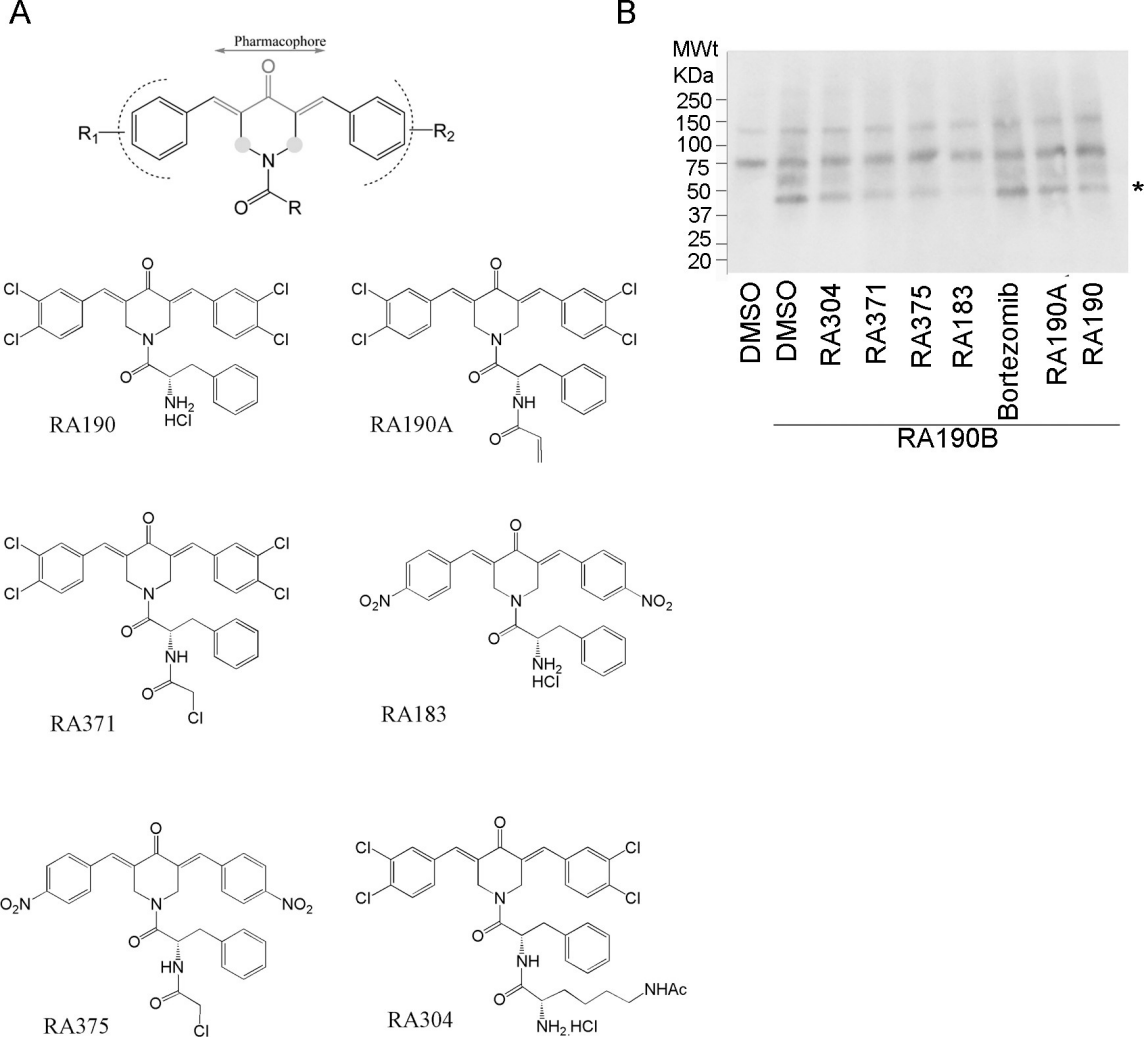
Impact of modifications around the RA190 core moiety. **(A)** Pharmacophore is denoted. R, R1 and R2 are modification sites to potentially alter the molecule’s physical and chemical properties while retaining the mechanism of action. Modifications at the green dots may allow the molecule to orient in one of four possible confirmations of cyclohexanone. **(B)** Precleared OV2008 cell lysate was incubated with compounds (20 μM) or vehicle (DMSO, 1:100) for 45 min and then with RA190B (10 μM) for 45 min at 4°C. Samples were subjected to SDS-PAGE, transfer to PVDF membrane, and binding of HRP-streptavidin, detected using chemiluminescence.

When our first series of 36 compounds (**S1 Table**) were tested using an XTT assay for their cytotoxicity against two human cell lines derived from a cervical cancer (HeLa) and an ovarian cancer (SKOV3), enhanced potency was evident with the addition of a war head to the free amine in RA190, with increased potency from H<COCH_3_<COCF_3_<CO-CH = CH_2_<CO-CH_2_Cl. This enhanced potency was not noticeably associated with increased aqueous solubility. Addition of a second charged amino acid, lysine, to the phenyl alanine of RA190 (compounds 17, 18 and 19 in **S1 Table**) achieved significant aqueous solubility but decreased potency. Compound 17 (RA310) achieved complete aqueous solubility with 3 fold decreased potency compared to RA190. Chloroacetamide-containing RA371 (compound 36 in **S1 Table and Fig 2A**) was the most potent molecule in this series. Next we examined the impact of substituents of the aromatic ring on the potency of molecules. The presence of substituents comprising either an electron withdrawing group (EWG) or an electron donating group (EDG) influences the reactivity of a Michael acceptor towards the target protein but can impact also impact specificity/selectivity. Compounds 37–49 (**S1 Table**) were synthesized using variety of EWG/EDG substituents on the aromatic ring. We chose the most promising substituents identified in the first series (COCH_3_<COCF_3_<CO-CH = CH_2_<CO-CH_2_Cl) to couple with core ring “N” and tested all the analogs for their activity against the two cancer cell lines. Molecules possessing substituents with stronger EWG (NO_2_>F>Cl>H>OMe>OH) effects were the most potent in the series. Remarkably, RA375 (compound 42 in **S1 Table and Fig 2A**) exhibited ten-fold greater activity against the two cancer lines than RA190, likely reflecting ring substituents with the strongest EWG and the potent chloroacetamide moiety. In a competition assay, pre-incubation of OV2008 cancer cell lysate with selected compounds, including RA375, abrogated the binding of RA190B to its 42KDa cellular target to varying degrees (**Fig 2B**) that were consistent with their IC50 values for cell killing (**S1 Table**).

Compounds containing hydroxyl substituents (43 and 44, **S1 Table**) exhibited complete aqueous solubility but 5–8 fold decreased potency compared to RA190. The impact on potency of replacing the aromatic ring with a small heterocycle and bulky groups was examined. Heterocycle replacement (Compounds 51 and 52, **S1 Table**) led to 3–4 fold decreased cytotoxic potency whereas the biphenyl replacement caused a 7 fold decrease in activity. Surprisingly extension of one unsaturated bond leads to 20–25 fold reduced activity (Compounds 54, 55, **S1 Table**). By contrast, the compounds possessing a chloroacetamide group, RA371 and RA375 (compounds 36 and 42 in **S1 Table**), were the most potent in the series. Although chloroacetamide is the active component of several candidate GSTO1 inhibitors [28, 29], in a competition assay using KT59 as a click chemistry probe [28], there was no evidence of RA375 competing its binding to GSTO1 (**S1A Fig**).

The cyclohexanone frame of these molecules tends to assume conformational isomerism which might negatively influence their properties. We speculated that making a bridge between two SP^3^ carbons of the cyclohexanone would confer a more restricted confirmation that might affect the reactivity of Michael acceptor (compounds 56–67, **S2 Table**) but bridge compounds exhibited one to two fold decreased cytotoxicity compared to without the bridge, and were not further pursued. Instead, RA371 and RA375 were selected for further studies.

### Effect of lead compounds on diverse cancer cell lines

We first examined the cytotoxic efficacy of selected compounds against multiple myeloma (MM) cell lines, as this is a validated clinical target for the licensed proteasome inhibitors. Cell lines were treated for 48 hr with 2-fold titrations of each compound (**S3 Table**) and their effect on proliferation compared to RA190, the structurally-related DUB inhibitors b-AP15 [31] and VLX1570 [32], using the MTT assay. The MM lines were most sensitive to RA375 treatment (**S3 Table**). Bortezomib-treated patients and cell lines typically develop resistance [33, 34]. When testing its potency against two MM cell lines that were selected for resistance *in vitro* by extended culture in bortezomib (V10R), RA375 is similarly efficacious against both the bortezomib-resistant derivative lines and their parental lines, consistent with a distinct mode of action from bortezomib (**S3 Table** and **S1B and S1C Fig**).

An initial survey of cytotoxicity for a panel of epithelial cancer cell lines suggested that ovarian, triple negative breast cancer (TNBC) and colon cancer cell lines were particularly sensitive to RA375 (**S3 Table**). RA375 showed similar potency in both a paclitaxel-resistant SKOV3 clone and its parental cell line (**S3 Table** and **S1D and S1E Fig**). RA375 was synergistic with doxorubicin in several ovarian cancer lines (**S3 Table**). Recent work suggested that the mutant TP53-driven TNBC subtype, like high grade ovarian cancer, is highly dependent on proteasome function [35] and RA375 showed significant potency against a small panel of TNBC lines. RA375 also potently inhibited TNBC and ovarian cancer cell colony formation (**S3A and S3B Fig**).

Cervical cancer is also a promising target because the HPV E6 oncoprotein drives transformation by proteasome-mediated degradation of key cellular targets, notably the p53. RA375 promoted the rapid onset of apoptosis in HPV16+ CaSki and SiHa and HPV18+ HeLa cells (**S3 Table**). The same phenomenon was clear in HPV+ head and neck cancer lines **(S1F Fig)**. Overall, HPV negative cervical and head and neck cancer lines, were ~4 fold less sensitive to RA375 than HPV+ lines, regardless of HPV genotype. Human pancreatic cancer-derived cell lines were substantially less sensitive to these compounds whether using MTT assay in either a 2D or 3D culture format (**S2 Fig**).

### Impact of glutathione metabolism on RA375 potency

**Fig 3:**
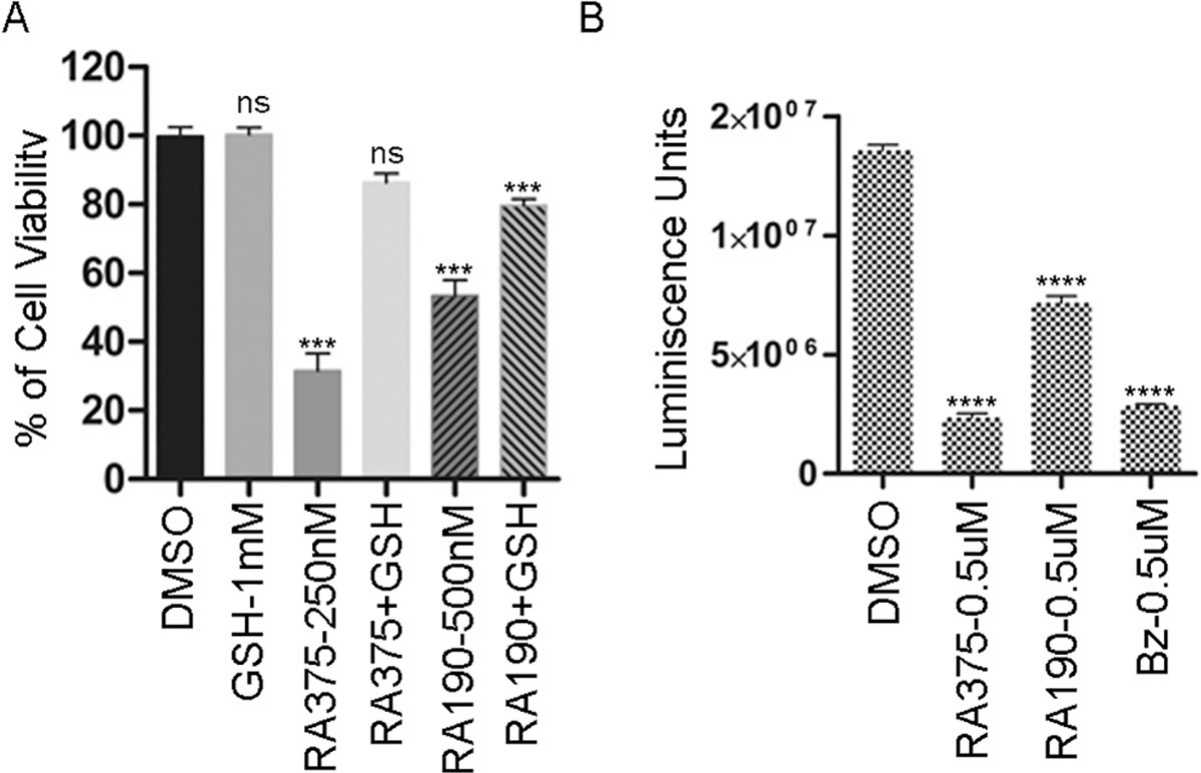
Effect of GSH on RA375 activity. **(A)** SKOV3 cells (2500 cells/well) were seeded in triplicate in a 96 well plate and one day later were treated with RA375 or RA190 in the presence or absence of GSH (1 mM). Cell viability was measured after 24 hrs using an MTT assay. Significance versus DMSO control (ns = not significant, *** <0.001, **** <0.0001. **(B)** SKOV3 cells (250,000/well) were treated in triplicate with DMSO vehicle alone or 0.5 μM RA375, RA190 or bortezomib (Bz) for 12 hr and the total GSH was measured.

To determine whether reaction of extracellular thiols impacts the potency of the RA371 and RA375, MTT assays were performed on SKOV3 cells either in the presence or absence of cysteine and methionine in the cell culture medium (although we utilized 10% FBS that had not been dialyzed to support cell viability). No significant difference in the activity of compounds in media with, versus without, cysteine and methionine was noted (**S3C–S3E Fig**). Addition of glutathione (GSH) to 0.5 mM in the SKOV3 cell culture medium reduced the cell killing effect of 250nM RA375 (p<0.001) and 500 nM RA190 (p<0.001) as compared to without (**Fig 3A**). Bortezomib profoundly reduced cellular GSH levels (**Fig 3B**) in SKOV3 cells (p<0.001) as previously reported in other cell types [26, 27]. Similarly, RA375, and to a lesser extent RA190 (each p<0.001), reduced intracellular GSH levels (**Fig 3B**), implying that these compounds may act in part by depleting GSH levels in cancer cells, and thus contribute to the ROS-associated cell killing effects in addition to their ER stress related to proteasome inhibition.

Loss of ARID1A function is a common driver of ovarian and some other cancer types, and it confers vulnerability to inhibition of GSH [36]. The parental HCT116 cell line was 4-fold less sensitive to RA375 than its isogenic *ARID1A* knockout, although this difference was less apparent for RA183, RA190 and bortezomib (**S3 Table**). There was no significant difference in the sensitivity of *ARID1A* wild type (OVCAR3, OVCAR5, ES2) and deficient (TOV21G, SKOV3, A2780) ovarian cancer lines to RA375 treatment (**S3 Table**) [37].

### Induction of unresolved ER stress, ROS and subsequent apoptosis by RA375

**Fig 4:**
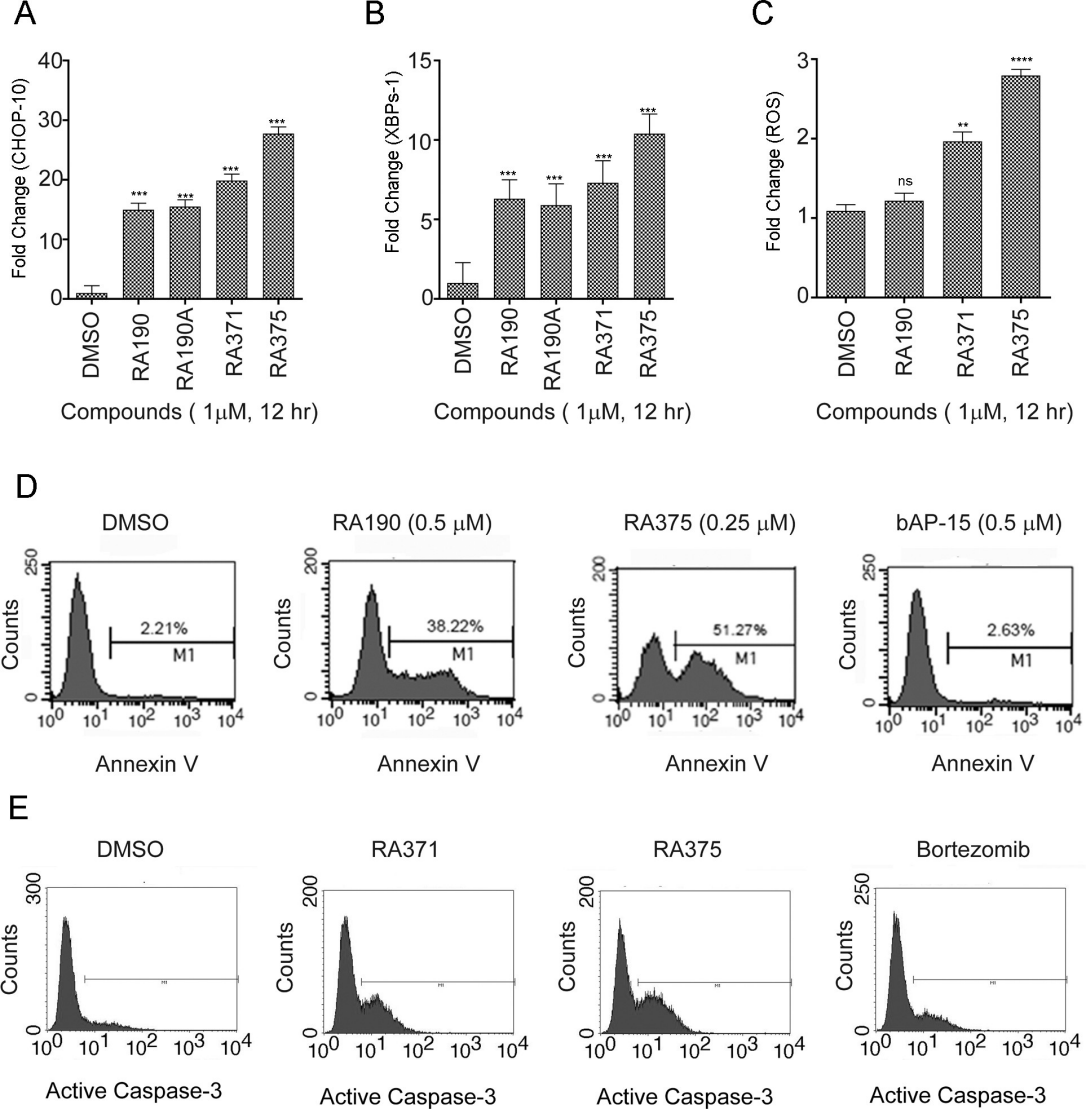
RA375 activates UPR signaling, ROS production and apoptosis. **(A-B)** ES2 cells (250,000 cells/well) were treated with compounds at 1 μM for 12 hr and mRNA was isolated. Samples were subjected to RT-qPCR to measure *CHOP10* **(A)** and *XBP-1s* **(B)** mRNA levels normalized to *GAPDH* expression. Significance versus DMSO control (ns = not significant, ** <0.01, *** <0.001, **** <0.0001). **(C)** ES2 cells (250,000 cells/well) were treated with compounds at 1 μM for 12 hr and incubated with H_2_DCFDA (20 μM) for 30 min and analyzed by flow cytometry. H_2_O_2_ was used as a positive control. For surface PS staining, 10^5^ ES2 cells treated with compounds for 12 hr were re-suspended in binding buffer and labeled with Annexin V-PE and 7-AAD at RT for 15 min and analyzed by flow cytometry **(D)** (Becton Dickinson, Mountain View, CA). **(E)** ES2 cells treated with either vehicle or compounds (1 μM, 18 hr) were fixed and probed with anti-Caspase 3 antibody and analyzed by flow cytometry.

Inhibition of proteasome function [10] triggers the unfolded protein response (UPR) and thereafter apoptosis independently of p53 signaling [38–40]. Early UPR-induced signaling is rapidly upregulated by RA375 treatment of ES2 cells including *CHOP-10* mRNA (**Fig 4A**) and *XBP*1 spliced mRNA (**Fig 4B**). RA371 and RA375 induced significantly higher levels of *CHOP-10* mRNA than RA190 (p<0.01 and p<0.001 respectively, **Fig 4A**), whereas only RA375 induced significantly higher levels of *XBP*1 spliced mRNA (p<0.001, **Fig 4B**). Since RA375 depleted cellular GSH (**Fig 3B**), reducing protection from oxidative stress, reactive oxygen species (ROS) were monitored by flow cytometry in cells treated with H_2_DCFDA which is cleaved to a fluorescent product by ROS, and treatment with H_2_O_2_ was used as a positive control. ROS were significantly induced in ES2 cells by RA371 and RA375 (**Fig 4C**), although only RA375 induced significantly higher levels than RA190 (p<0.01). Similar results were seen in SKOV3 cells, and RA375 induced higher levels of ROS than RA190 or bortezomib (**S4A Fig**). Unresolved UPR and ROS activate apoptosis in cancer cells, and annexin V-cell surface labeling was detected 12 hr after treatment of ES2 cells with 0.5μM RA190 or 0.25μM RA375 resulted in 38% and 50% cells undergoing apoptosis respectively (**Fig 4D**). A similar phenomenon was observed with SKOV3 cells (**S4B Fig**). Treatment of ES2 cells with RA371 and RA375 also induced rapid cleavage of Caspase 3, an apoptosis marker (**Fig 4E**). Since RA375 is more potent *in vitro*, it was advanced for preliminary murine studies.

### Safety and pharmacodynamics of RA375

Initial toxicity testing was performed in female Balb/c mice with RA375. Groups of mice (n = 3) were injected intra peritoneally (*i*.*p*.) with a single dose of RA375 (5, 10, 20, 40, 60, 100 mg/Kg in 25% β-hydroxypropyl cyclodextrin) and endpoints evaluated included clinical observation and body weight for one week. No adverse effects were noted with RA375 at even the highest dose. Administration of RA375 (40 mg/Kg, n = 5) on alternate days for two weeks produced no observable toxicities or weight loss.

**Fig 5:**
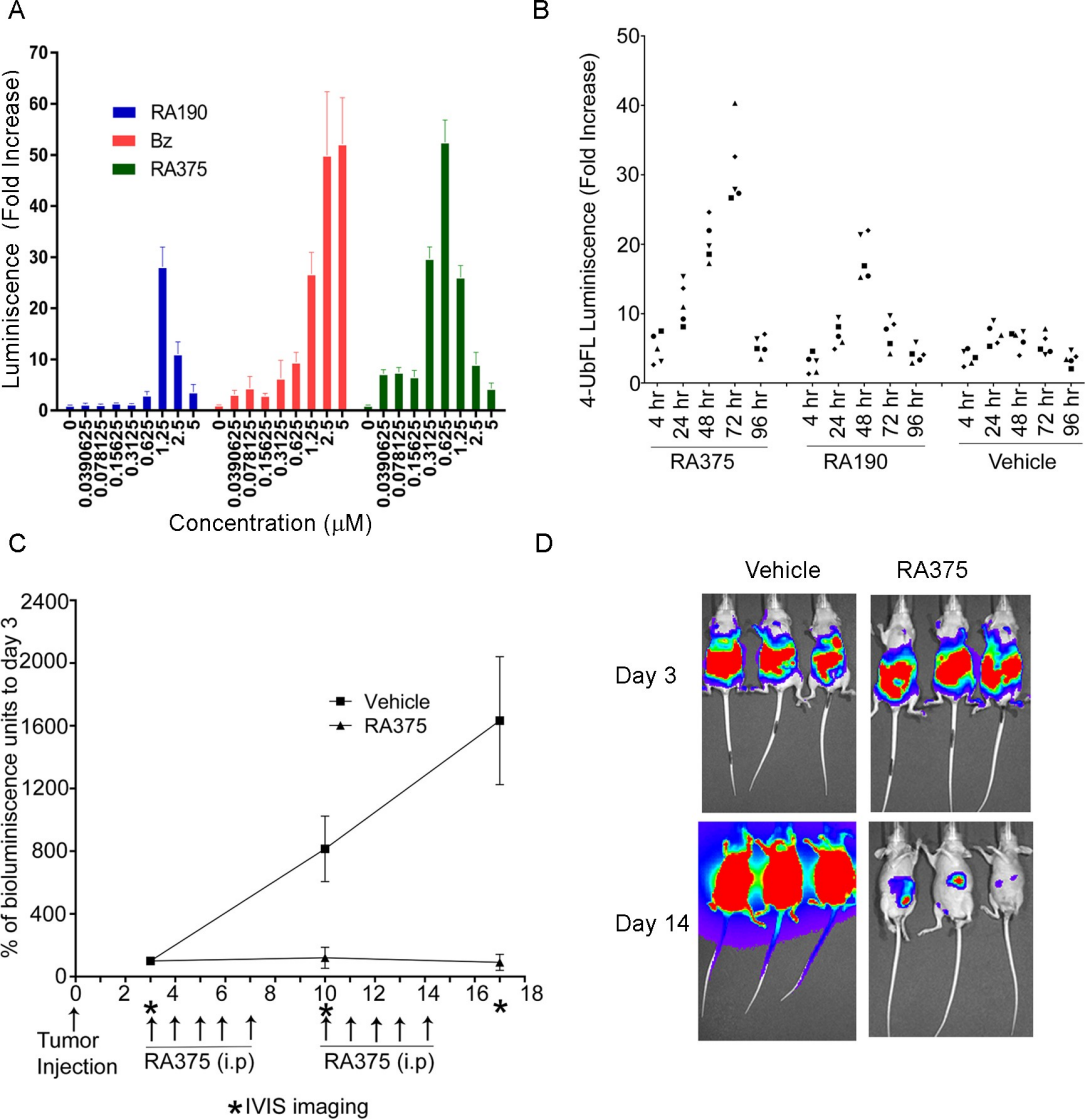
RA375 inhibits proteasome function and reduces ovarian tumor burden in mice. **(A)** 293T cells in 96 well plates were transiently transected with the 4UbFL plasmid and 48 hr later treated with the indicated doses of each compound for 4 hr. Cells were lysed and luciferase activity assessed using a luminometer. **(B)** BALB/c mice were electroporated with 4UbFL plasmid (10 μg/mouse) in the leg muscle and the basal luminescence was recorded after 48 hr. Groups of mice (n = 5) were treated with different compound (40 mg/Kg) or vehicle alone (25% (*w*/*v*) β-Hydroxypropylcyclodextrin in water). Mice were imaged for bioluminiscence activity using IVIS instrument at 4, 24, 48, 72 and 96 hr. **(C-D)** Nude mice (8 per group) were inoculated with 10^6^ ES2-luc cells *i*.*p*. in 100 μL PBS. Three days later the mice were imaged for basal level (day 0) bioluminescence expression using an IVIS200. Mice were randomized into two groups (n = 8) and treated daily i.p. with RA375 (10 mg/Kg) or vehicle (25% (*w*/*v*) β-Hydroxypropylcyclodextrin) for a 5 days on, 2 days off cycle for two weeks and imaged again on day 7 and day 14 for tumor burden.

To monitor proteasome activity in living cells, we used a construct which expresses a fusion protein comprising four tandem repeats of ubiquitin fused in frame with firefly luciferase (4UbFL) [41] to cause this otherwise stable reporter protein to undergo rapid proteolytic degradation via the proteasome. To assess their capacity to inhibit cellular proteasome function, 293T cells transiently transfected with the 4UbFL construct and then 24h later treated with compounds for 4 hours prior to assay of luciferase activity. Like bortezomib, RA375 induced a dramatic increase in bioluminescence, consistent with stabilization of 4UbFL, in a dose dependent manner. At very high concentrations the luciferase activity dropped because of cellular toxicity. RA375 was 2-fold more potent than bortezomib and 4-fold than RA190 (**Fig 5A**).

To test for proteasome inhibition by RA375 *in vivo*, we first employed an electroporation delivery to transfect the leg muscle of mice with the 4UbFL reporter plasmid. After *i*.*p*. injection of luciferin, the enzymic activity of luciferase expressed by the 4UbFL DNA vector in the muscle tissue was visualized as bioluminescence using an IVIS imager. At two days post electroporation of the 4UbFL DNA, mice were imaged and base line luminescence recorded. The control group (n = 5) of mice was treated *i*.*p*. with vehicle alone and additional groups (n = 5) treated *i*.*p*. with single 40 mg/Kg doses of RA375 or RA190 (**Fig 5B**). After 4h, 24h, 48h, 72h and 96 h post treatment mice were again imaged and bioluminescence was quantified. Stronger increases in bioluminescence were observed after treatment with RA375 than RA190 indicating greater inhibition of proteasome function *in vivo* (**Fig 5B**).

### Therapeutic effect of RA375 on human xenograft model of clear cell ovarian carcinoma

ES2 is a human cell line derived from a high grade ovarian clear cell carcinoma [42]. The efficacy of RA375 against the ES2 xenograft model expressing luciferase (ES2-luc) was tested. Nude female mice were inoculated with ES2-luc cells into the peritoneal cavity. After 3 days the mice were imaged for their basal luminescence activity and randomized into two groups (n = 8). One group was treated with the vehicle alone (25% β-hydroxypropyl-cyclodextrin solution in water) and the other group treated with RA375 (10mg/Kg) daily for 5 days on treatment, two off, for 2 weeks. Mice were imaged after the first and second week of treatment for their luciferase activity to assess tumor burden. RA375 significantly reduced tumor burden (**Fig 5C and 5D**) without apparent weight loss or side effects and extended survival (p = 0.04; **S5 Fig**).

## Discussion

This study provides insight into the structural requirements necessary for drug binding to RPN13, presumably by adducting Cys-88 [6, 12, 13], and informs design of molecules more potent than our prototype RA190 [10]. The flexibility of the core unit allows numerous modifications around the pharmacophore without disturbing target specificity. Building upon our previous findings with our RA- chemical series [10, 13, 43–45], and related inhibitors [19], herein we have identified important structure-activity relationships and analogs with a second warhead (RA371 and RA375) were identified as having increased potency. It is noteworthy that the related RPN13 inhibitors CLEFMA and EF24 have also demonstrated therapeutic activity against preclinical cancer models via related mechanisms [19].

Cancer cells critically differ from normal cells in higher metabolic rate, ROS and aberrant protein synthesis [46, 47]. Proteasomes play a pivotal role managing this excessive metabolism and maintaining protein homeostasis, as the continued over-accumulation of mis-folded proteins is toxic to cells via UPR-induced apoptosis, thereby providing a therapeutic window for proteasome inhibitors [47]. Several proteasome inhibitors have proven efficacious against hematologic malignancies, although failure later due to resistance, significant neurological side effects and inactivity against solid tumors remain challenges. The PSMB5-targeted proteasome inhibitors are developed from polypeptide backbones or large natural compounds that may not readily enter solid tumors. Indeed, bortezomib exhibited limited activity against *s*.*c*. ES2 xenografts [48] and in patients against ovarian cancer [49–55] suggesting the need for proteasome inhibitors with a different backbone that better access solid tumor tissues.

The licensed proteasome inhibitors each target one of the three proteolytic functions located in the 20S catalytic subunit, the chymotrypic activity of PSMB5. This redundancy of proteasome inhibition function may also explain how these proteasome inhibitors are tolerated by normal cells and the host. The two receptors RPN10 and RPN13 on the 19S RP co-operate in recognizing ubiquitinated proteins that are targeted for degradation [56]. In mice, liver-specific deletion of either RPN10 or RPN13 produced limited effects, but simultaneous loss of both RPN10 and RPN13 caused severe liver injury accompanied by massive accumulation of ubiquitin conjugates [57]. This is consistent with cooperative roles of RPN10 and RPN13, and therefore some redundancy, in ubiquitin recognition of the proteasome. Furthermore, several additional ubiquitin receptors have been described for the 19S RP, such as DSS1 and RAD23A/B, also suggesting considerable redundancy [56]. Likewise, while RPN13 promotes UCH37 activity, an additional DUB enzyme USP14 also plays an important role in deubiquitinating substrate proteins, thus potentially explaining the tolerability of systemic administration of RPN13 inhibitors. RPN13 was identified as a target of RA190 by adding a biotin tag for labeling studies (RA190B), but unfortunately this strategy is not available for RA371 and RA375 without compromising the pharmacophore. Since it was not possible to utilize this approach to determine whether the additional warhead promotes reaction with other cysteines in RPN13 or reactivity with other cellular targets, future studies should address this issue. Nevertheless, these compounds did compete RA190B binding to RPN13 in cell lysates.

RPN13 is an unusual proteasome component in that under normal conditions a single molecule binds to either one end or the other of the proteasome to direct the asymmetric degradation of a polyubiquitinated substrate rather than simultaneous processing from both ends [58]. Since RPN13 is over expressed in cancer [17], Kisselev has hypothesized that the covalent RA inhibitors block both ends of the proteasome, whereas in normal cells only one end is block and the other can utilize alternative ubiquitin receptors to maintain some proteasome function [14]. However, recent structural data suggest that these RA inhibitors act to prevent RPN2 binding and thus association with the 19S RP [4, 59].

The ubiquitin chain topology determines how ubiquitination establishes precise communication in cells. While K48-linked ubiquitinated proteins are tagged for degradation, K63, K33 and K11 linked proteins are tagged for cell cycle maintenance and activation/in-activation of signaling pathways and DNA damage repair [56]. While the RA compounds caused accumulation of K48-linked substrates, this was not the case for K63-linked polyubquitinated proteins (not shown).

In conclusion, we have sought to make more potent RPN13 inhibitors utilizing a rational development approach to judiciously modify substituents around the core unit of RA190 and, after several rounds of this, RA375 emerged as a promising compound based on pharmacodynamics and its reduction of tumor burden and prolongation of the survival of mice carrying an orthotopic human ovarian cancer xenograft. Since bortezomib has not proven effective against ovarian and other solid cancers, further exploration of this new class of RPN13 inhibitors, potentially in combination with doxorubicin, is warranted because their novel mechanism of action.

## Supporting information

S1 Table IC_50_ of compounds 1–57 against HeLa and SKOV3 cells. (DOCX)

S2 Table IC_50_ of bridge compounds 58–70 against HeLa and SKOV3 cells. (DOCX)

S3 Table IC_50_ values (nM) of compounds for cell lines derived from diverse cancer types and normal tissues. (DOCX)

S4 Table Synergy scores of RA183 or RA375 in combination with approved chemotherapeutic agents. (DOCX)

S1 Methods Synthesis and characterization of RA371 and RA375. (DOCX)

S1 Fig Impact of RA375 on GSTO1 binding by KT59 and cancer cell viability. **(A)** In this approach the alkyne moiety of KT59 is reacted with fluorescent azide reporter via click chemistry to reveal inhibitor-labeled proteins. SKOV3 cell lysate was treated with 10 μM of KT59 for 30 min at 25°C. For competition with RA190 and RA375, lysate was first treated withRA190 or RA375 at indicated concentrations for 45 min at 4°C prior to addition of KT59 (10 μM, 30 min) Cell lysate was boiled in Laemmli buffer and separated by SDS-PAGE, and transferred to PVDF membrane. Next membrane was treated with Alexa Fluor 488 azide (5 μL, Cat. No. A10266, Life Technologies) for 45 min at room temperature in the presence of CuSO_4_ (10 μL of 10 mM stock) and sodium ascorbate (20 μL of 20 mM stock) in PBST (10 mL). Membrane was washed with PBST (3 times for 20 min) and blocked with 1% BSA for 1 hr and then probed with antibody for Alexa488 (Rabbit polyclonal, Life Technologies, Cat No. A-11094) in 1% BSA in PBST for 1 hr. Membrane was washed with PBST for 3 times and incubated with secondary antibody in PBST for 1 hr and washed with PBST (3X for 20 min) and developed using chemiluminiscence reagent by Biorad Imager. **(B-C)** Multiple Myeloma cell line RPMI8226 and its bortezomib resistant version (RPMI-8226-V10R) were treated with either DMSO or RA375 **(B)** or bortezomib **(C)** for 48 hr and the cell viability was compared using MTT. **(D-E)** Ovarian cancer cell line SKOV3 and its paclitaxel resistant version (SKOV3-TR) were treated with either DMSO, RA375 **(D)** or paclitaxel **(E)** for 48 hr and the cell viability was assayed using MTT **(F)** A panel of cell lines derived from HPV positive and negative cervical cancers as well as head and neck cancers were treated with RA375 for 48 hr and the cell viability was compared using MTT.
(TIF)

S2 Fig Effect of compounds against pancreatic cancer cell growth. A panel of pancreatic cancer cell lines (Panc 10.05, Panc 215 and A6L) growing in 2D culture (**left**) as compared to 3D culture (**right**) were measured at 48 hr after growth in the presence of compounds at indicated concentrations. For 2D killing assays, 5000 cells/well were plated in a 96 well plate in 50μL medium. After 24 hr cells were treated with compounds in 50μL medium and incubated at 37°C for 96 hr. After the incubation medium was removed, 0.2% SDS was added (50μL/well) and incubated at 37°C for 2hrs. Then 150μL of SYBR Green I solution (1:750 in water) was mixed with the cell lysate, and the fluorescence measured using FLUOstar-Galaxy plate reader. For 3D killing assays, 3000 cells/well seeded in a 384 well plate (Corning spheroid microplate, cat No. 3830) in 25 μL medium. After confirming spheroid formation (200–400 μm) at day 3, drug solutions (25 μL) were added to corresponding wells. At day 6, 10% SDS (5 μL) was added to each well followed by 50μL of cell-titer-glo reagent. The microplate was vigorously mixed for 2 min on an orbital shaker to induce cell lysis and release cellular ATP, 100 μL transferred to a white flat bottom 384-well plate (Sigma 460372). After briefly centrifuging the plate to remove bubbles and the ATP quantification was measured using a Wallac 1420 multi label counter.
(TIF)

S3 Fig Impact of RA190, RA371 and RA375 on clonogenicity, cell viability and levels and size of polyubiqutinated proteins. **(A-B)** HS578T **(A)** or SKOV3 cells **(B)** were plated at 300/well in 2 mL DMEM growth medium in a 6 well plate and incubated at 37°C for a day. Cells were treated with compounds at the indicated doses and incubated for 14 days to allow colony formation. The plates were stained with 1% crystal violet in methanol and clusters containing 50 or more cells were scored as a colony. **(C-E)** SKOV3 cells grown in 10% FCS/DMEM medium lacking methionine and cysteine were compared with cells grown in standard DMEM for 48 hr in the presence of compounds. Cell viability was measured using an MTT assay.
(TIF)

S4 Fig Activation of ROS production and apoptosis by compounds. **(A)** SKOV3 cells were treated for 12 hr with compounds (or as a positive control, H_2_O_2_) at the indicated doses and ROS levels were measured by adding Amplex Red and HRP. **(B)** To analyze apoptosis, 10^5^ SKOV3 cells were treated with compounds (1μM, 12 hr), then re-suspended in 100 μL binding buffer with 5 μL of Annexin V-PE and 5 μL of 7-AAD. After a 15 min incubation at RT, the cells were analyzed by flow cytometry using a FACSCalibur and CellQuest software (Becton Dickinson).
(TIF)

S5 Fig RA375 treatment enhances survival of mice bearing ES2-luc xenograft. The experiment was performed as described in Fig 5C and 5D and the survival data was presented using Kaplan-Meier analysis and the statistical significance by the log rank test.
(TIF)

S6 Fig Raw images. Raw images of Fig 1B probed with HRP-streptavidin, Fig 1C probed with anti-ubiquitin, Fig 1C probed with anti-actin, Fig 2B probed with HRP-streptavidin, and **S1**A Fig probed with KT59 are presented in this order. Position of markers is indicated in pen.
(PDF)
