# Supplementary material for: Structure-function analyses of candidate small molecule RPN13 inhibitors with antitumor properties
Source: PLoS One. 2020 Jan 15;15(1):e0227727. doi: 10.1371/journal.pone.0227727 (PMC6961910; doi:10.1371/journal.pone.0227727)
Supplement: S1 Table — (DOCX) [file pone.0227727.s001.docx]

Table S1. IC_50_ of compounds 1-57 against HeLa and SKOV3 cells.

|  | | | | |  |  |
| --- | --- | --- | --- | --- | --- | --- |
| S.No | Code | R1 | R2 | P | HeLa (nM) | SKOV3 (nM) |
| 1 | RA7 |  |  | H | 532 | 654 |
| 2 | RA190 |  |  |  | 85 | 75 |
| 3 | RA190  ACR |  |  |  | 42 | 62 |
| 4 | RA190Ac |  |  |  | 46 | 68 |
| 5 | RA190P |  |  |  | 55 | 64 |
| 6 | RA190SA |  |  |  | 121 | 253 |
| 7 | RA190NMS |  |  |  | 78 | 94 |
| 8 | RA190IAS |  |  |  | 211 | 342 |
| 9 | RA190Bn |  |  |  | 86 | 121 |
| 10 | RA190PyC |  |  |  | 65 | 84 |
| 11 | RA232 |  |  |  | 61 | 77 |
| 12 | RA230 |  |  |  | 47 | 69 |
| 13 | RA233 |  |  |  | 76 | 98 |
| 14 | RA234 |  |  |  | 143 | 235 |
| 15 | RA231 |  |  |  | 111 | 167 |
| 16 | RA190VA |  |  |  | 74 | 87 |
| 17 | RA310 |  |  |  | 543 | 446 |
| 18 | RA310Ac |  |  |  | 195 | 284 |
| 19 | RA195Bn |  |  |  | 146 | 171 |
| 20 | RA190CA |  |  |  | 51 | 84 |
| 21 | RA190TMB |  |  |  | 94 | 112 |
| 22 | RA190MU |  |  |  | 141 | 239 |
| 23 | RA190AHA |  |  |  | 62 | 88 |
| 24 | RA190VBA |  |  |  | 94 | 178 |
| 25 | RA228 |  |  |  | 66 | 98 |
| 26 | RA7-Val |  |  |  | 64 | 72 |
| 27 | RA7-His |  |  |  | 132 | 166 |
| 28 | RA221 |  |  |  | 43 | 59 |
| 29 | RA295 |  |  |  | 89 | 116 |
| 30 | RA195 |  |  |  | 109 | 132 |
| 31 | RA7-Gly |  |  |  | 65 | 78 |
| 32 | RA7-Ala |  |  |  | 68 | 92 |
| 33 | RA7-Ile |  |  |  | 72 | 101 |
| 34 | RA7-IleC |  |  |  | 61 | 77 |
| 35 | RA190MS |  |  |  | 86 | 133 |
| 36 | RA371 |  |  |  | 44 | 47 |
| 37 | RA-asy |  |  | H | 312 | 398 |
| 38 | RA-asyP |  |  |  | 48 | 62 |
| 39 | RA183Acr |  |  |  | 49 | 64 |
| 40 | RA183Ac |  |  |  | 56 | 71 |
| 41 | RA183CF3 |  |  |  | 45 | 79 |
| 42 | RA375 |  |  |  | 13 | 26 |
| 43 | DHBC |  |  | H | 667 | 788 |
| 44 | HBC |  |  | H | 453 | 531 |
| 45 | RA-CTF |  |  | H | 324 | 457 |
| 46 | RA-CTFP |  |  |  | 56 | 81 |
| 47 | RA181 |  |  |  | 63 | 89 |
| 48 | RA-4Cl |  |  |  | 71 | 83 |
| 49 | RA-TMP |  |  |  | 78 | 125 |
| 50 | RA-BT |  |  | H | 454 | 642 |
| 51 | RA-BTP |  |  |  | 212 | 243 |
| 52 | RA-BTPCA |  |  |  | 132 | 189 |
| 53 | RA-biP |  |  | H | 1432 | 1786 |
| 54 | RA-Cinn |  |  | H | >5000 | >5000 |
| 55 | RA181C |  |  |  | >2500 | >2500 |
| 56 | RA190H |  | | | 562 |  |
| 57 | RA190B |  | | | 346 |  |
