# Supplementary material for: Structure-function analyses of candidate small molecule RPN13 inhibitors with antitumor properties
Source: PLoS One. 2020 Jan 15;15(1):e0227727. doi: 10.1371/journal.pone.0227727 (PMC6961910; doi:10.1371/journal.pone.0227727)
Supplement: S2 Table — (DOCX) [file pone.0227727.s002.docx]

Table S2. IC_50_ of bridge compounds 58-70 against HeLa and SKOV3 cells.

|  | | | | | HeLa | SKOV3 |
| --- | --- | --- | --- | --- | --- | --- |
| 58 | RA330 |  |  | H | 239 | 278 |
| 59 | RA336 |  |  | CO=CH=CH2 | 146 | 185 |
| 60 | RA337 |  |  | COCH3 | 186 | 231 |
| 61 | RA335 |  |  | COCH3 | 161 | 251 |
| 62 | RA329 |  |  | H | 407 | 471 |
| 63 | RA338 |  |  |  | 66 | 81 |
| 64 | RA334 |  |  | CO=CH=CH2 | 101 | 186 |
| 65 | RA339 |  |  |  | 46 | 54 |
| 66 | RA331 |  |  | H | 236 | 301 |
| 67 | RA333 |  |  | CO=CH=CH2 | 112 | 167 |
| 68 | RA332 |  |  | H | 243 | 376 |
| 69 | RA340 |  |  | CO=CH=CH2 | 184 | 259 |
| 70 | RA342 |  |  |  | 122 | 181 |
