# Supplementary material for: Structure-function analyses of candidate small molecule RPN13 inhibitors with antitumor properties
Source: PLoS One. 2020 Jan 15;15(1):e0227727. doi: 10.1371/journal.pone.0227727 (PMC6961910; doi:10.1371/journal.pone.0227727)
Supplement: S3 Table — (DOCX) [file pone.0227727.s003.docx]

Table S3. IC_50_ values (nM) of compounds for cell lines derived from diverse cancer types and normal tissues.

| Cancer Type | Cell Lines | Compounds | | | |
| --- | --- | --- | --- | --- | --- |
|  |  | RA190 | RA183 | RA375 | Bortezomib |
| Cervical  (HPV +ve) | HeLa | 85 | 112 | 13 |  |
|  | SiHa | 603 | 263 | 41 |  |
|  | CaSki | 324 | 312 | 43 |  |
|  | ME180 | 183 | 293 | 20 |  |
| Cervical  (HPV -ve) | HT3 |  |  | 162 |  |
|  | C33A |  |  | 167 |  |
| Head & Neck  (HPV +ve) | SSC90 |  |  | 24 |  |
|  | VU147T |  |  | 40 |  |
| Head & Neck  (HPV -ve) | SSC25 |  |  | 189 |  |
|  | JHU11 |  |  | 178 |  |
|  | JHU29 |  |  | 173 |  |
|  | FaDu |  |  | 142 |  |
| Ovarian | OVCAR3 | 120 | 78 | 17 |  |
|  | OVCAR5 | 64 | 56 | 12 |  |
|  | ES2 | 115 | 75 | 19 |  |
|  | TOV21G | 83 | 44 | 6 |  |
|  | SKOV3 | 73 | 54 | 26 |  |
|  | SKOV3-TR | 109 | 77 | 22 |  |
|  | A2780 | 139 | 161 | 37 |  |
|  | ID8 (murine) | 211 | 198 | 44 |  |
| Colon | HCT116 | 239 | 193 | 89 | 3.2 |
|  | HCT116  ARID1A^-/-^ | 164 | 155 | 20 | 2.8 |
| Multiple Myeloma | MM.1S | 72 | 46 | 11 |  |
|  | RPMI8226 | 45 | 88 | 9 |  |
|  | RPMI8226-V10R | 47 | 139 | 6 |  |
|  | ANBL6 | 67 | 58 | 12 |  |
|  | ANBL6-V10R | 55 | 103 | 16 |  |
| Prostate | LNCaP | 242 | 186 | 60 |  |
|  | PC3 | 162 | 133 | 27 |  |
| Triple Negative Breast | HS578T | 78 | 89 | 3 |  |
|  | MDA-MB231 | 134 | 124 | 22 |  |
|  | HCC1806 | 52 | 44 | 17 |  |
| Human Foreskin  Fibroblast | HFF | >1000 | >1000 | >1000 | >100 |
| Mouse Skin | Keratinocytes | >1000 | >1000 | >1000 | >100 |
