# Supplementary material for: Structure-function analyses of candidate small molecule RPN13 inhibitors with antitumor properties
Source: PLoS One. 2020 Jan 15;15(1):e0227727. doi: 10.1371/journal.pone.0227727 (PMC6961910; doi:10.1371/journal.pone.0227727)
Supplement: S4 Table — (DOCX) [file pone.0227727.s004.docx]

Table S4. Synergy scores of RA183 or RA375 in combination with approved chemotherapeutic agents.

| Synergy Scores | | | |  |  |
| --- | --- | --- | --- | --- | --- |
| **Compounds** | **Cell Line** | **HSA** | **Loewe** | **Bliss** | **ZiP** |
| Cisplatin+RA183 | OVCAR3 | 17.954 | 16.101 | 16.127 | 16.561 |
| Doxorubicin+RA183 | OVCAR3 | 17.775 | 15.183 | 15.206 | 15.6 |
| Vincristine+RA183 | OVCAR3 | 8.732 | 5.618 | 3.841 | 3.975 |
| Doxorubicin+RA375 | ID8 | 14.201 |  | 6.083 | 6.08 |
| Doxorubicin+RA375 | SSC90 | 9.141 | 5.326 | 4.423 | 4.481 |
| Doxorubicin+RA375 | OVCAR3 | 10.66 |  | 10.46 | 10.51 |
