# Supplementary material for: Structure-function analyses of candidate small molecule RPN13 inhibitors with antitumor properties
Source: PLoS One. 2020 Jan 15;15(1):e0227727. doi: 10.1371/journal.pone.0227727 (PMC6961910; doi:10.1371/journal.pone.0227727)
Supplement: S1 Methods — (DOCX) [file pone.0227727.s005.docx]

**Synthesis and characterization of RA375:**

RA183 (0.63g) dissolved in DCM was added to 3 eq of DIPEA and 1.1 eq of chloroacetyl chloride at 0°C. The reaction mixture was stirred at room temperature for 3 hr, and saturated sodium bicarbonate solution was added. Excess DCM was added and the layers were separated. The organic layer was washed with H_2_O and brine and dried over Na_2_SO_4_. The solvent was removed in vacuo and the residue was purified by column chromatography on silica gel eluted with ethyl acetate: hexanes to obtain RA375 with >97% purity confirmed by NMR and MS.


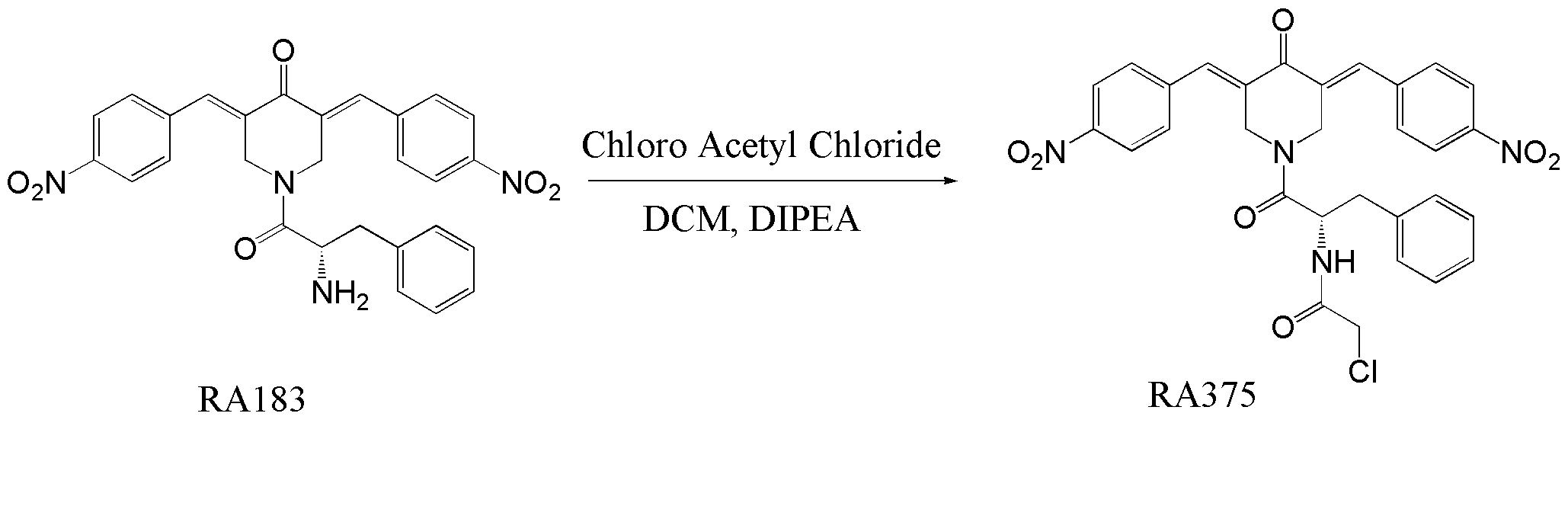
yield, 0.47 g; ^1^H NMR (400 MHz, CDCl_3_): 2.8-2.94 (m, 2 H), 3.98 (s, 2H), 4.18-4.55 (dd, 2H), 4.72-4.99 (m, 3H), 6.98-7.05 (m, 2H), 7.22-7.47 (m, 5H), 7.63-7.67 (m, 2H), 7.71-7.82 (m, 2H), 8.28-8.45 (m, 4H); M+ 592.

**Synthesis and Characterization of RA371:**

RA190 (0.54g) dissolved in DCM was added to 3 eq of DIPEA and 1.1 eq of chloroacetyl chloride at 0°C. The reaction mixture was stirred at room temperature for 3 hr and saturated sodium bicarbonate solution was added. Excess DCM was added and the layers were separated. The organic layer washed with H_2_O and brine and dried over Na_2_SO_4_. The solvent was removed in vacuo and the residue was purified by column chromatography on silica gel eluted with ethyl acetate: hexanes to obtain RA371 with >97% purity confirmed by NMR and MS.


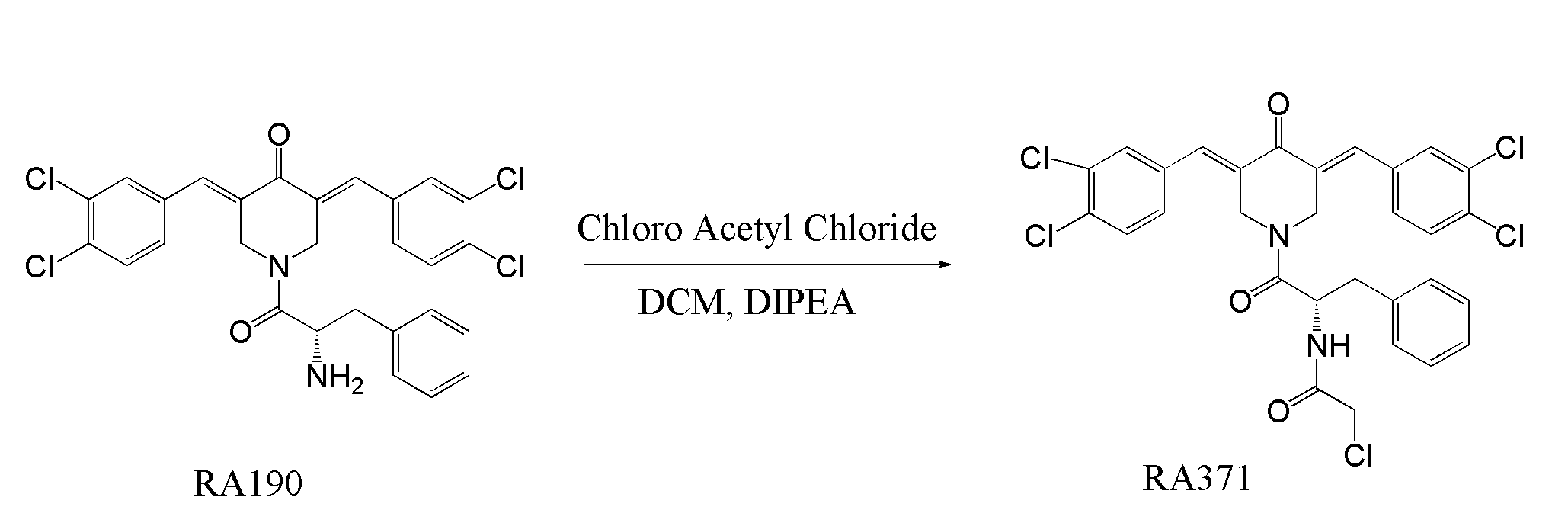
yield, 0.37 g; ^1^H NMR (400 MHz, CDCl_3_): 2.78-2.97 (m, 2 H), 3.99 (s, 2H), 4.12-4.17 (m, 1H), 4.43-4.78 (dd, 2H), 4.8-4.99 (m, 2H), 6.99-7.20 (m, 3H), 7.23-7.47(m, 6H), 7.52-7.69 (m, 4H).
