# Supplementary figures and images for: Structure-function analyses of candidate small molecule RPN13 inhibitors with antitumor properties
Source: PLoS One. 2020 Jan 15;15(1):e0227727. doi: 10.1371/journal.pone.0227727 (PMC6961910; doi:10.1371/journal.pone.0227727)

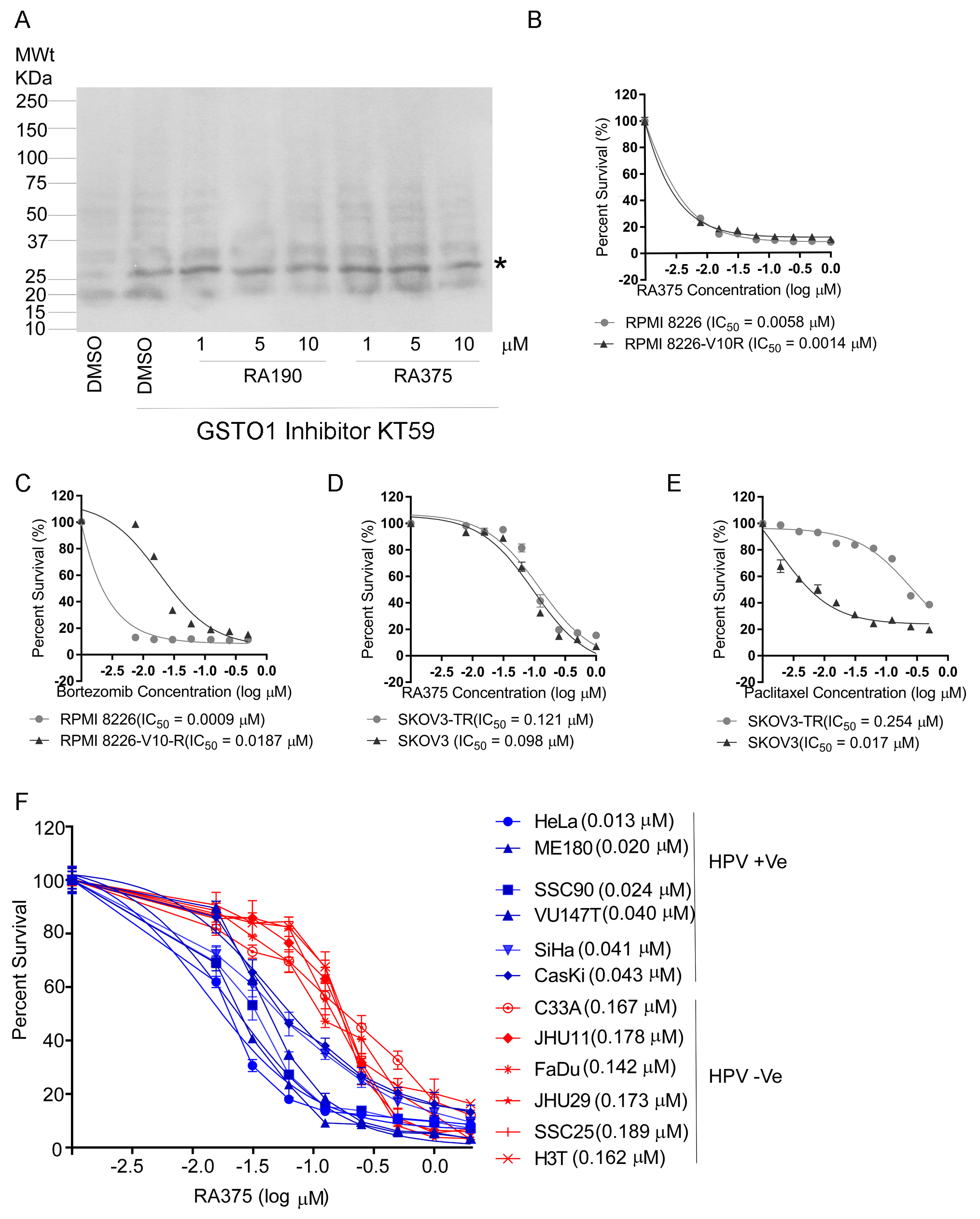

Supplement: S1 Fig — (A) In this approach the alkyne moiety of KT59 is reacted with fluorescent azide reporter via click chemistry to reveal inhibitor-labeled proteins. SKOV3 cell lysate was treated with 10 μM of KT59 for 30 min at 25°C. For competition with RA190 and RA375, lysate was first treated withRA190 or RA375 at indicated concentrations for 45 min at 4°C prior to addition of KT59 (10 μM, 30 min) Cell lysate was boiled in Laemmli buffer and separated by SDS-PAGE, and transferred to PVDF membrane. Next membrane was treated with Alexa Fluor 488 azide (5 μL, Cat. No. A10266, Life Technologies) for 45 min at room temperature in the presence of CuSO4 (10 μL of 10 mM stock) and sodium ascorbate (20 μL of 20 mM stock) in PBST (10 mL). Membrane was washed with PBST (3 times for 20 min) and blocked with 1% BSA for 1 hr and then probed with antibody for Alexa488 (Rabbit polyclonal, Life Technologies, Cat No. A-11094) in 1% BSA in PBST for 1 hr. Membrane was washed with PBST for 3 times and incubated with secondary antibody in PBST for 1 hr and washed with PBST (3X for 20 min) and developed using chemiluminiscence reagent by Biorad Imager. (B-C) Multiple Myeloma cell line RPMI8226 and its bortezomib resistant version (RPMI-8226-V10R) were treated with either DMSO or RA375 (B) or bortezomib (C) for 48 hr and the cell viability was compared using MTT. (D-E) Ovarian cancer cell line SKOV3 and its paclitaxel resistant version (SKOV3-TR) were treated with either DMSO, RA375 (D) or paclitaxel (E) for 48 hr and the cell viability was assayed using MTT (F) A panel of cell lines derived from HPV positive and negative cervical cancers as well as head and neck cancers were treated with RA375 for 48 hr and the cell viability was compared using MTT. (TIF) [file pone.0227727.s006.tif]

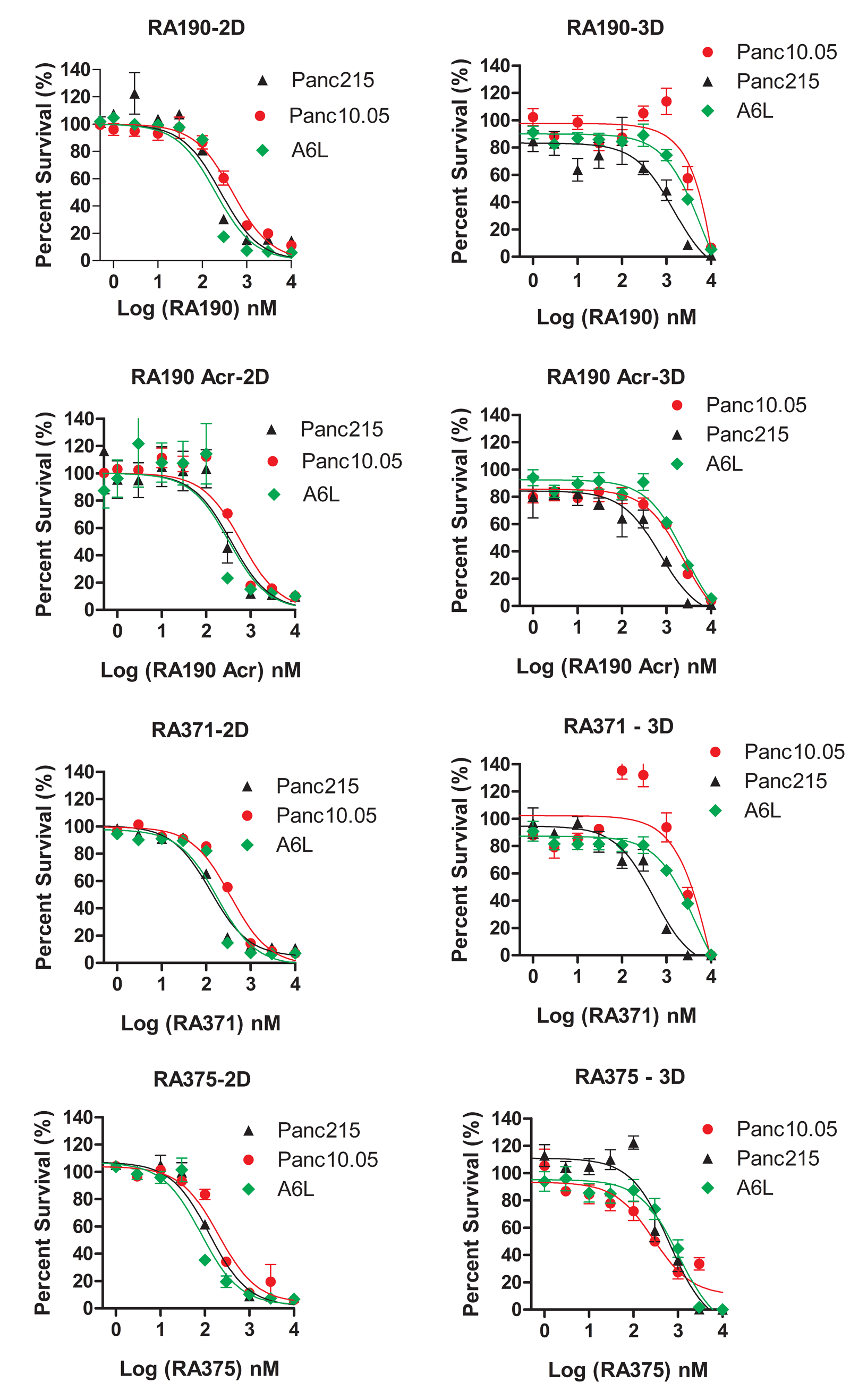

Supplement: S2 Fig — A panel of pancreatic cancer cell lines (Panc 10.05, Panc 215 and A6L) growing in 2D culture (left) as compared to 3D culture (right) were measured at 48 hr after growth in the presence of compounds at indicated concentrations. For 2D killing assays, 5000 cells/well were plated in a 96 well plate in 50μL medium. After 24 hr cells were treated with compounds in 50μL medium and incubated at 37°C for 96 hr. After the incubation medium was removed, 0.2% SDS was added (50μL/well) and incubated at 37°C for 2hrs. Then 150μL of SYBR Green I solution (1:750 in water) was mixed with the cell lysate, and the fluorescence measured using FLUOstar-Galaxy plate reader. For 3D killing assays, 3000 cells/well seeded in a 384 well plate (Corning spheroid microplate, cat No. 3830) in 25 μL medium. After confirming spheroid formation (200–400 μm) at day 3, drug solutions (25 μL) were added to corresponding wells. At day 6, 10% SDS (5 μL) was added to each well followed by 50μL of cell-titer-glo reagent. The microplate was vigorously mixed for 2 min on an orbital shaker to induce cell lysis and release cellular ATP, 100 μL transferred to a white flat bottom 384-well plate (Sigma 460372). After briefly centrifuging the plate to remove bubbles and the ATP quantification was measured using a Wallac 1420 multi label counter. (TIF) [file pone.0227727.s007.tif]

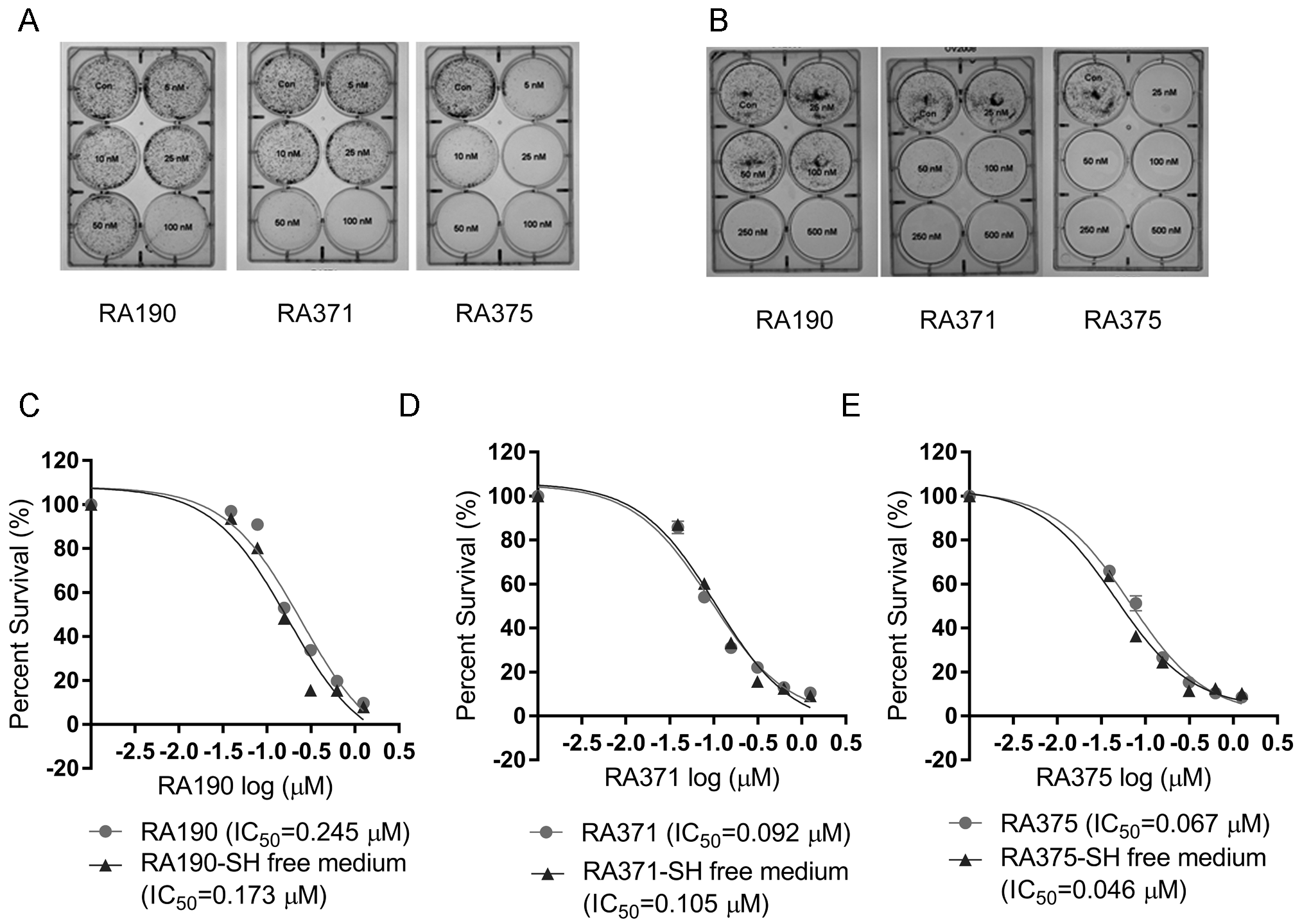

Supplement: S3 Fig — (A-B) HS578T (A) or SKOV3 cells (B) were plated at 300/well in 2 mL DMEM growth medium in a 6 well plate and incubated at 37°C for a day. Cells were treated with compounds at the indicated doses and incubated for 14 days to allow colony formation. The plates were stained with 1% crystal violet in methanol and clusters containing 50 or more cells were scored as a colony. (C-E) SKOV3 cells grown in 10% FCS/DMEM medium lacking methionine and cysteine were compared with cells grown in standard DMEM for 48 hr in the presence of compounds. Cell viability was measured using an MTT assay. (TIF) [file pone.0227727.s008.tif]

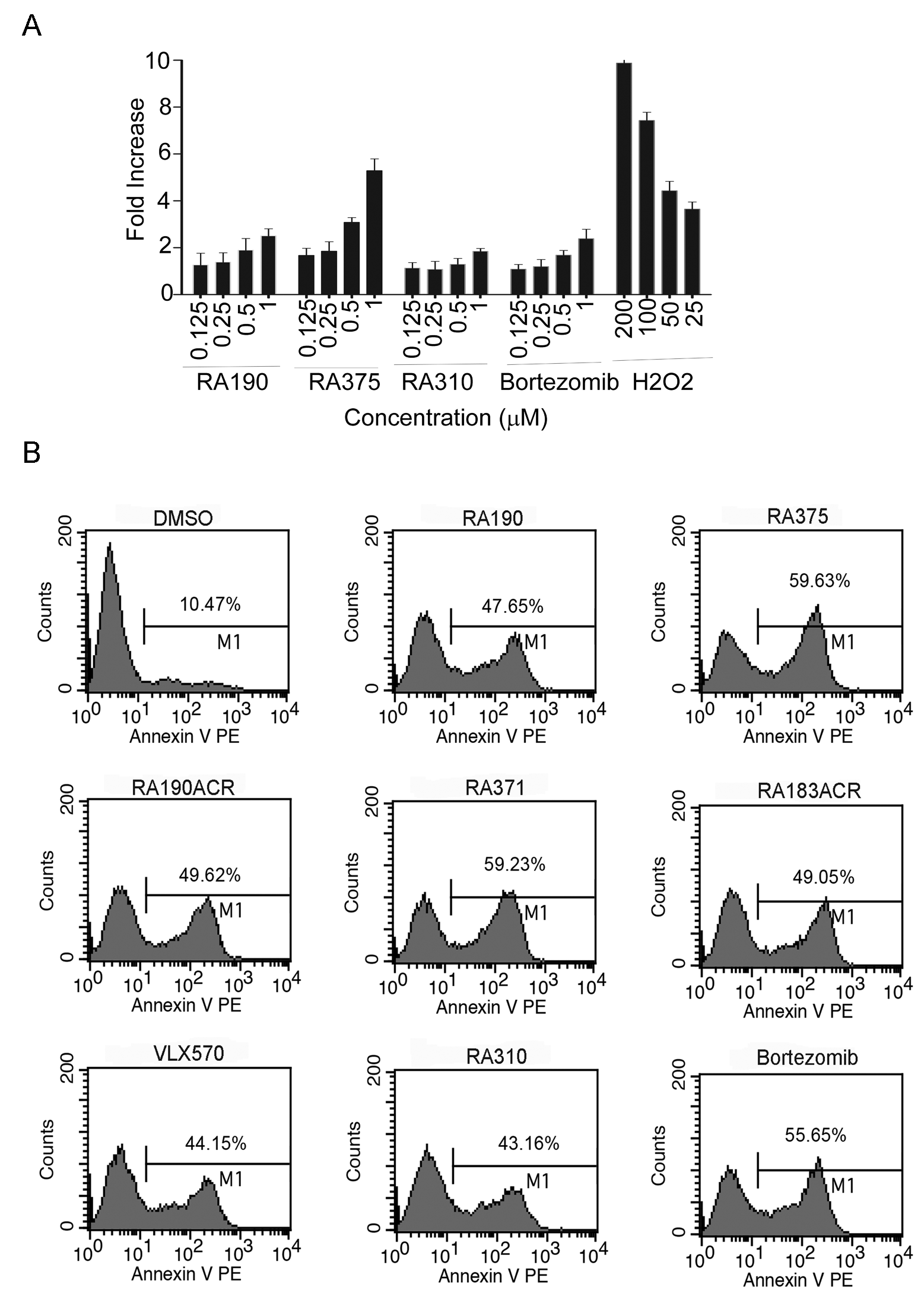

Supplement: S4 Fig — (A) SKOV3 cells were treated for 12 hr with compounds (or as a positive control, H2O2) at the indicated doses and ROS levels were measured by adding Amplex Red and HRP. (B) To analyze apoptosis, 105 SKOV3 cells were treated with compounds (1μM, 12 hr), then re-suspended in 100 μL binding buffer with 5 μL of Annexin V-PE and 5 μL of 7-AAD. After a 15 min incubation at RT, the cells were analyzed by flow cytometry using a FACSCalibur and CellQuest software (Becton Dickinson). (TIF) [file pone.0227727.s009.tif]

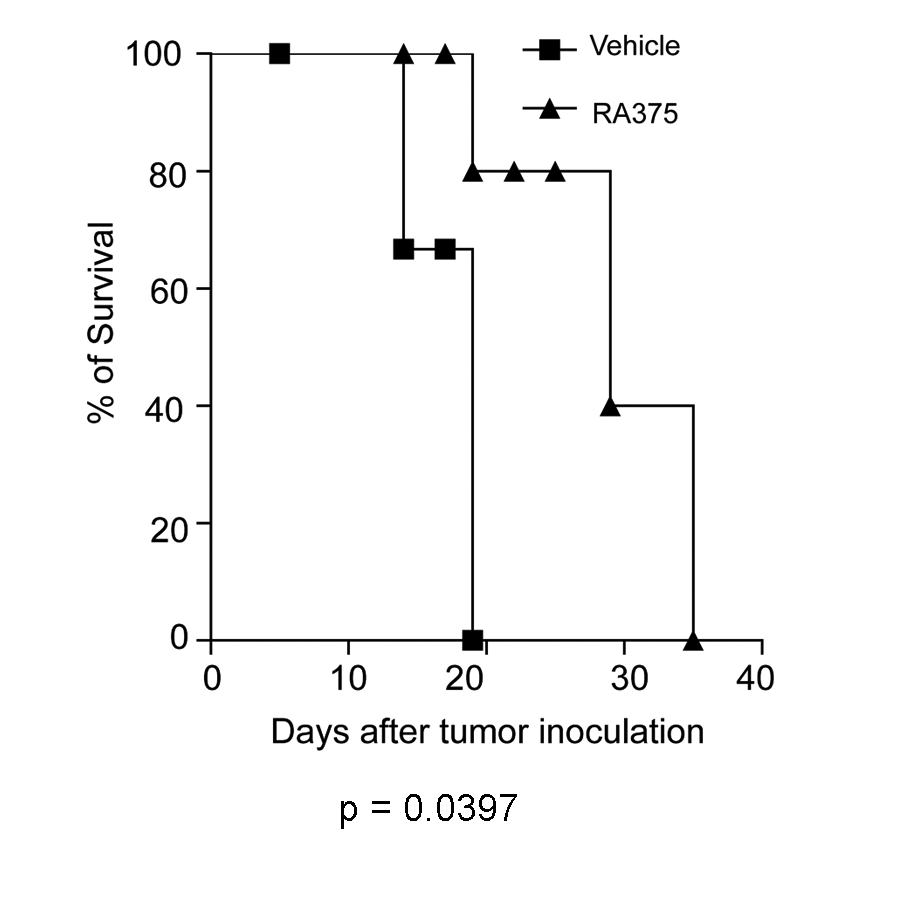

Supplement: S5 Fig — The experiment was performed as described in Fig 5C and 5D and the survival data was presented using Kaplan-Meier analysis and the statistical significance by the log rank test. (TIF) [file pone.0227727.s010.tif]

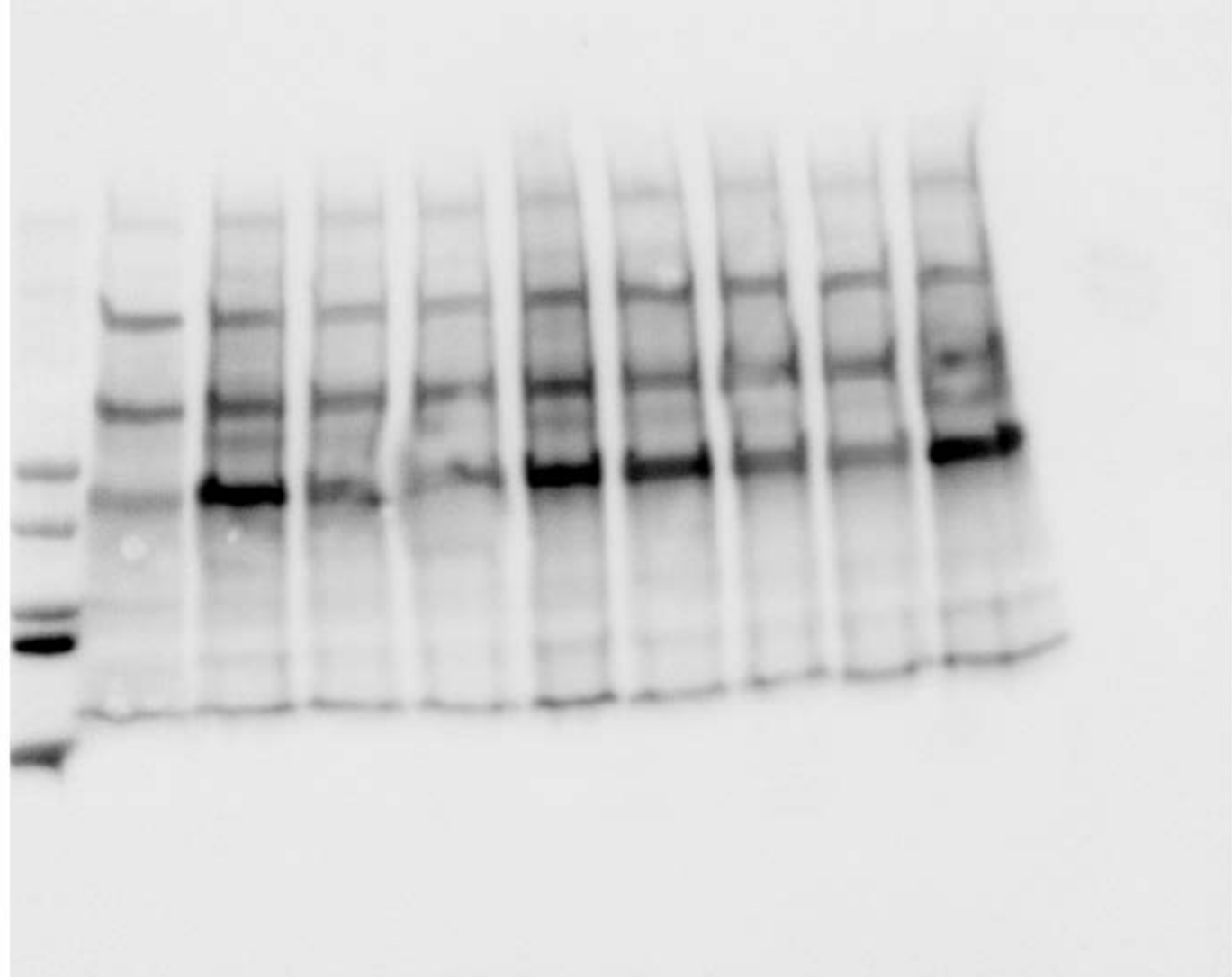

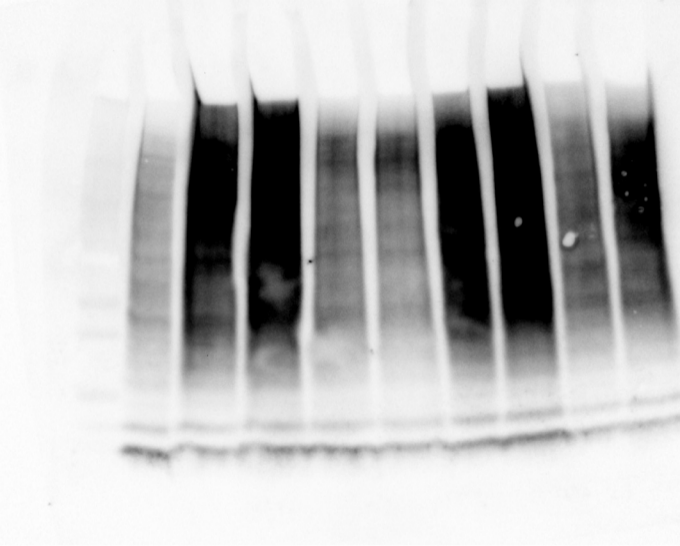

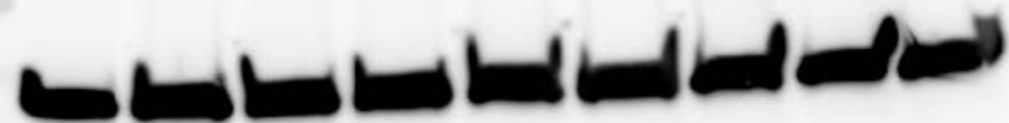

一一一

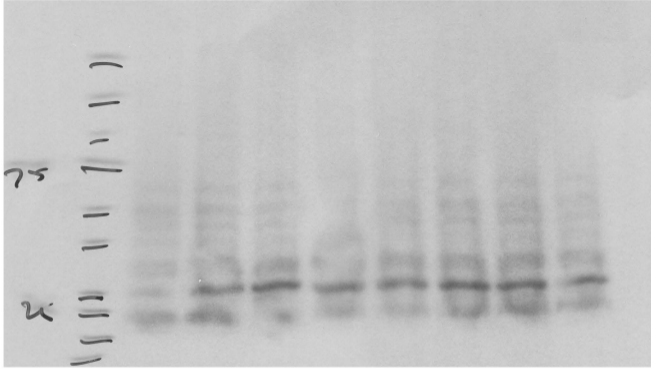

Supplement: S6 Fig — Raw images of Fig 1B probed with HRP-streptavidin, Fig 1C probed with anti-ubiquitin, Fig 1C probed with anti-actin, Fig 2B probed with HRP-streptavidin, and S1A Fig probed with KT59 are presented in this order. Position of markers is indicated in pen. (PDF) [file pone.0227727.s011.pdf]
